# Review of New Caledonian species of *Oxyethira* Eaton, with description of 17 new species, and new records for *Hydroptila* Dalman and *Hellyethira* Neboiss (Trichoptera, Hydroptilidae)

**DOI:** 10.3897/zookeys.530.6047

**Published:** 2015-10-28

**Authors:** Alice Wells, Kjell Arne Johanson

**Affiliations:** 1 Australian Biological Resources Study, PO Box 787, Canberra, ACT 2601 Australia; 2 Zoology Department, Swedish Museum of Natural History, Box 50007, SE-104 05 Stockholm, Sweden

**Keywords:** Spicipalpia, Hydroptilidae, New Caledonia, endemic, key, new species

## Abstract

New Caledonian representation of the cosmopolitan genus *Oxyethira* Eaton is reviewed, with the description of new species bringing to 26 the total for the genus on the island. The species are referred to three subgenera: *Trichoglene* Neboiss (11 species), *Pacificotrichia* Kelley (13 species) and *Dampfitrichia* Ulmer (one species) and one species is unplaced to subgenus. A key is provided to *Oxyethira* species of New Caledonia. In addition, new records are given for two otherwise Australian species, *Hydroptila losida* Mosely and *Hellyethira malleoforma* Wells. Points marked on a series of small maps of New Caledonia indicate the site or sites at which the species were collected. This final paper in a series of generic revisions brings the hydroptilid fauna of the island of New Caledonia to 60 species, distributed in six genera.

## Introduction

The cosmopolitan genus *Oxyethira* Eaton exhibits a diverse array of male genital structures and arrangements. The species show some variability in female terminalia, but exhibit extreme conservatism in larval and case morphology. Representatives of the genus found in New Caledonia appear to be no exception. This is apparent upon consideration of the variability among 26 species recorded here, and the similarity of the considerable number of unassociated final instar larvae collected at many sites by Mary (2002) in her survey of macroinvertebrates of the island’s streams.

This final paper in a series of genus-level reviews (Wells and Johanson 2012, 2014; Wells, Johanson and Mary-Sasal 2013) brings to 60 species, in six genera, the presently known hydroptilid fauna of New Caledonia, only surpassed by Ecnomidae in number of species found on the island (Espeland and Johanson 2010). The study is based for the greater part on collections of Hydroptilidae made by members of the Swedish Museum of Natural History and deals primarily with the genus *Oxyethira*. It also presents new records for the previously reported (Wells 1995) sole New Caledonian representatives of two other hydroptilid genera, *Hydroptila losida* Mosely, 1953 and *Hellyethira malleoforma* Wells, 1979, both common in eastern Australia.

*Oxyethira* is well represented in New Caledonia, at genus level only surpassed in known species diversity among the island Trichoptera by the helicopsychid genus *Helicopsyche* (Johanson 1999; Johanson and Mary 2000), with 30 described species, and the ecnomid genus *Agmina* (Espeland & Johanson, 2010), with 28 described and nearly 50 undescribed species. Seven New Caledonian *Oxyethira* species were described by Kelley (1989), based on a collection in the B.P. Bishop Museum, Honolulu. He assigned the species to two subgenera: subgenus *Trichoglene* Neboiss (*Oxyethira caledoniensis* Kelley and *Oxyethira insularis* Kelley); and a new subgenus, *Pacificotrichia* Kelley (*Oxyethira oropedion* Kelley, *Oxyethira dorsennus* Kelley, *Oxyethira indorsennus* Kelley, *Oxyethira melasma* Kelley, and *Oxyethira scutica* Kelley), all assigned to the “*oropedion* group”). Oláh and Johanson (2010a) described three additional New Caledonia species: *Oxyethira tompa*, which they referred to subgenus *Pacificotrichia*; and two species, *Oxyethira arok* and *Oxyethira derek*, which they assigned to subgenus *Trichoglene*. One representative of a third subgenus, *Dampfitrichia* Ulmer, the widespread SE Asian-Australasian species Oxyethira (Dampfitrichia) incana (Ulmer) described from Java, is recorded from New Caledonia for the first time.

Most of the 17 species newly described here from New Caledonian can be referred to the above three subgenera with a degree of confidence. One species, however, cannot be placed at present: *Oxyethira macropennis* sp. n. shares the diagnostic features of *Oxyethira* as defined by Kelley (1984) and is left unplaced.

Among females in the samples at least two general morphological forms can be recognised in abdominal terminalia: a short oviscapt of the form illustrated by Kelley (1989: figs 55, 56) for the Vanuatuan *Oxyethira efatensis* Kelley; and a slender, elongate oviscapt such as he illustrated for *Oxyethira oropedion* (Figs 50, 51) and *Oxyethira scutica* (Figs 52, 53), sometimes with a small, rounded black area ventrally on abdominal segment X as in *Oxyethira oropedion*. The distinctive female of *Oxyethira incana* is readily recognised by the quadrate black patch ventrally on segment X. Some of the other females were associated with males tentatively, but we are not sufficiently confident of their identity to include them here.

Apparent distributions of species are difficult to interpret (see Figs 86–113). Almost all collections were made during November to mid January, normally the warmer season of the year. At least over that period, some species appear to be very localised, others widespread and still others disjunct in distribution, being taken from far northern and far southern localities. Further studies are needed at other times of year to determine whether these data reflect reality, seasonality, or some aspect of behaviour, such that collecting methods missed particular species.

## Methods

Most of the material this study is based upon was collected in light traps and Malaise traps situated near or across running water. Specimens were prepared for study as Canada balsam slide mounts following maceration in KOH and clearing in clove oil. Male genitalia are illustrated in line drawings, traced from draft figures using Adobe Illustrator CS5, for species for which suitable slides are available.

An identification key and descriptions of New Caledonian *Oxyethira* species are provided, as well as brief diagnoses of previously described *Oxyethira* species and new illustrations of their male genitalia, drawn from types and/or newly collected non-type specimens. Species descriptions are based on male genital features, although identification of homologies among these is often difficult, especially for some of the more aberrant species. Usually diagnostic features are indicated on figures. Terms applied to genital structures follow the recommendations of Oláh and Johanson (2010b) and itemised by Wells and Johanson (2014: 3) when reviewing New Caledonian species of the genus *Acritoptila*.

Development of a useful key for easy identification of species was difficult as observation of most readily diagnostic features requires preparation of slide mounts of specimens and examination under a compound microscope.

Collection sites for species were plotted on a series of maps (Figs 86–113). Specimens in this study are deposited in the following repositories:

MNHP Muséum National d’Histoire Naturelle, Paris, France

NHRS Swedish Museum of Natural History, Stockholm, Sweden

ANIC Australian National Insect Collection, CSIRO, Canberra, Australia

BPBM Bishop Museum, Hawaii, USA

## Systematics

### *Oxyethira* Eaton

#### Subgenus *Trichoglene* Neboiss

The chief diagnostic characteristics that Kelley (1989) notes for subgenus *Trichoglene* are: in males, “a complete non-excised [abdominal] segment VIII”, identified as plesiomorphic, and “aedeagus with recurved sub-distal spinous process and subgenital processes widely separated and partly fused with each pleuron of segment IX”, features identified as apomorphic. Additional features are included in Kelley’s description of the subgenus, including a titillator on the “aedeagus” [= phallic apparatus]. The subgenital processes in most members of this subgenus are in the form of a pair of well-separated rods, spines or strap-like structures, connected basally with the gonopods (= inferior appendages of Kelley) and a pair of digitiform membranous lobes, each bearing an apical seta.

In discussions of subgenus *Trichoglene* (Kelley 1984, 1989), some confusion is evident in understanding of the type species of *Trichoglene*. Neboiss (1977) established the genus *Trichoglene* for *Trichoglene columba* Neboiss, described from Tasmania. This species was recognised by Wells (1981) as a species of *Oxyethira*. Upon designation of *Trichoglene* as a sugenus of *Oxyethira*, Kelley (1984) incorrectly gave the New Zealand *Oxyethira albiceps* (McLachlan, 1862) (= *Hydroptila albiceps* McLachlan, 1862) as the type. He repeated and compounded the error (Kelley 1989) by stating that “the type species of *Trichoglene* was incorrectly identified as *Oxyethira columba* (Neboiss) in Kelley (1984) [which it is not]… [i]t should be *Oxyethira albiceps* (MacLachlan)’. Perhaps he meant to imply that *Hydroptila albiceps* and *Trichoglene columba* (= *Oxyethira columba*) are synonyms, but they are distinct. *Trichoglene columba* Neboiss is the type species of the subgenus. Subgenus *Trichoglene* is Australasian in distribution, occurring in Australia, including Tasmania and Norfolk Island, and in New Zealand, as well as New Caledonia. Among New Caledonian species, Kelley assigned *Oxyethira caledoniensis* and *Oxyethira insularis* to subgenus *Trichoglene*; and eight species are newly described here.

Three species groups are recognised among these New Caledonian members of subgenus *Trichoglene*. A set of species, the *spinifera*-group, with abdominal segment IX subquadrate comprises *Oxyethira spinifera* sp. n., *Oxyethira tiwaka* sp. n., *Oxyethira perignonica* sp. n., and *Oxyethira abbreviata* sp. n. A second set, the *caledoniensis*-group, with venter of abdominal segment IX in ventral view produced anteriorly, proximally either rounded or tapered and somewhat triangular, includes *Oxyethira caledoniensis* Kelley, *Oxyethira incurvata* sp. n., *Oxyethira arok* Oláh & Johanson, *Oxyethira amieu* sp. n., and *Oxyethira houailou* sp. n. The distinctions between these two groups of species, however, are not clear cut. The venter of *Oxyethira caledoniensis* Kelley is only slightly produced anteriorly. A third group, the *insularis*-group, characterised by the prominently Y-shaped gonopods has only two members: *Oxyethira insularis* sp. n. and *Oxyethira parinsularis* sp. n.

##### Oxyethira (Trichoglene) spinifera sp. n.

Taxon classificationAnimaliaTrichopteraHydroptilidae

http://zoobank.org/EC7AC788-3322-4798-88A2-1F1C876C5FF1

Figs 1–3 , 86

###### Diagnosis.

Males closely resemble those of Oxyethira (Trichoglene) tiwaka sp. n. and Oxyethira (Trichoglene) perignonica sp. n. in having abdominal segment IX subquadrate in ventral view, with the apico-ventral margin truncate but both those species have recognisable gonopods, albeit strongly reduced, whereas as in Oxyethira (Trichoglene) arok, Oxyethira (Trichoglene) amieu and Oxyethira (Trichoglene) spinifera gonopods are so severely reduced that no gonopods can be identified. The rods of ventral processes of Oxyethira (Trichoglene) spinifera are slender and spiny, rather sharper than in Oxyethira (Trichoglene) tiwaka sp. n. but in both species the rods are almost parallel whereas in Oxyethira (Trichoglene) perignonica sp. n. they are sharply pointed and convergent.

###### Description.

Male antennae with 23–25 flagellomeres, flagellomeres rectangular in profile, without *sensilla placodea*, terminal 3 flagellomeres pale, rest dark; anterior wing length 1.3–1.5 mm (n=8); tibial spurs 0,3,4. Female antennae with 18 flagellomeres, flagellomeres all dark; anterior wing length 1.3–1.6 mm (n=4); tibial spurs 0,3,4. Abdominal sternite VII with sharp median spur.

**Figures 1–12.**
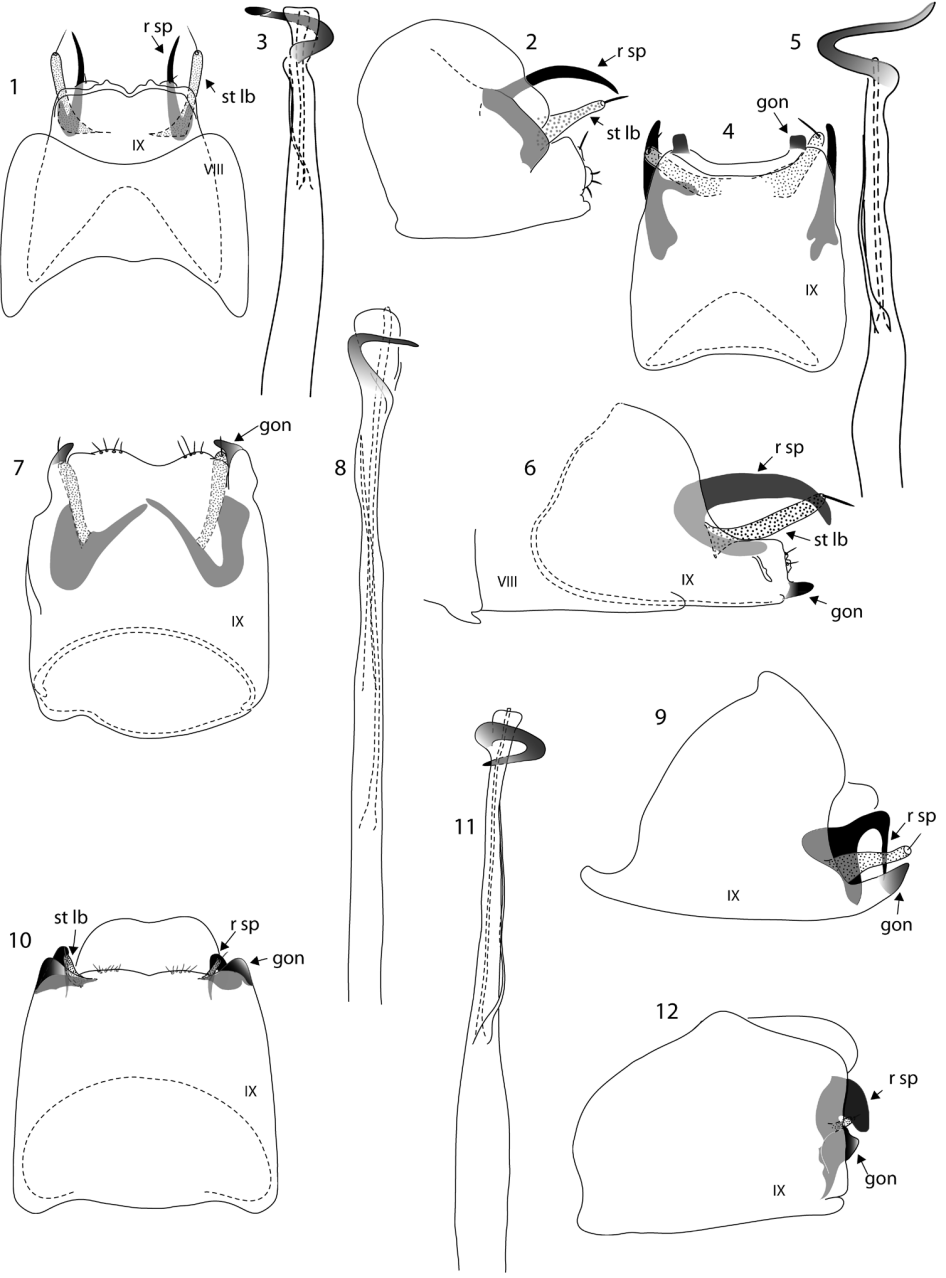
*Oxyethira* species male genitalia. **1–3** *Oxyethira spinifera* sp. n., ventral and lateral views, and phallic apparatus **4–6** *Oxyethira tiwaka* sp. n., ventral view, phallic apparatus and lateral view **7–9** *Oxyethira perignonica* sp. n., ventral view, phallic apparatus and lateral view **10–12** *Oxyethira abbreviata* sp. n., ventral view, phallic apparatus and lateral view. Abbreviations: gon = gonopod; r sp = rod of subgenital process; st lb = setose lobe of subgenital process; VIII, IX = abdominal segments VIII and IX.

Male, genitalia (Figs 1–3). Abdominal segment VIII in ventral view: width exceeds length; margins concave proximally and distally. Abdominal sternite IX: truncate distally. Gonopods reduced, not recognisable on apical margin. Rods of subgenital processes widely separated, parallel, in form of sharp spines. Phallic apparatus elongate, with slender titillator, and subapical narrow sinuous spine.

###### Material examined.

**Holotype.** Male (on slide), New Caledonia, small fall ~10 km SW Houailou on Houailou-Bourail road, 26.xii.1998, leg. A. Wells (MNHP).

**Paratypes.** 2 males (on slides), small stream ~15 km SW Houailou on Houailou–Bourail road, 26.xii.1998, leg. A. Wells (ANIC); 1 male (on slide), stream, ~20 km SW Thio on Boulouparis–Thio road, 28.xii.1998, leg. A. Wells (NHRS); 1 male, Province Sud, Monts Kwa Ne Mwa, on road between Noumea and Yaté, 2.0 km E Pic Mouirange, 22°12.356'S, 166°40.798'E, 220 m, 7–16.xi.2003, Malaise trap, loc#014, leg. K.A. Johanson (NHRS); 15 males, Province Sud, W slope Mt Ningua, Kwé Néco, Stream, at Camp Jacob, 3.7 km WNW summit of Mt Ningua, on Boulouparis–Thio Road, about 50 m upstream road, 21°43.613'S, 166°06.567'E, 150 m, 29.xi–12.xii.2003, Malaise trap, loc#054, leg. K.A. Johanson (NHRS); 24 males, 13 females (3 on slides), Province Nord, Wemwâdiu stream, 850 m E summit Kögi Mtn, 5 m upstream road, about 200 m S Tiwaka River, 20°49.020'S, 165°14.165'E, 24 m, 6–27.xii.2003, Malaise trap, loc#067, leg. K.A. Johanson (NHRS).

###### Additional material.

12 males, 6 females, Province Sud, Sarraméa, 2907 m, stony forest stream, loc 13, 21°37.097'S 165°49.351'E, Malaise trap, 18–21.xi.2001, leg. K.A. Johanson, T. Pape & B. Viklund (NHRS); 1 male, 1 female, Province Sud, Sarraméa, 220 m, forest stream, loc 10, 21°37.883'S 165°51.958'E, Malaise trap, 18–21.xi.2001, leg. K.A. Johanson, T. Pape & B. Viklund (NHRS); 5 males, 4 females, Province Sud, Monts des Koghis, ca 300 m S Koghi Restaurant, 22.18288°S, 166.50167°E, 417 m, 2–16.xi.2003, Malaise trap, loc#004, leg. K.A. Johanson (NHRS); 18 males, 10 females, Province Sud, Monts des Koghis, ca 800 m S Koghi Restaurant, 22.18311°S, 166.50564°E, 460 m, 10–26.xi.2003, Malaise trap, loc#019, leg. K.A. Johanson (NHRS); males, females, Province Sud, W slope Mt Ningua, Kwé Néco Stream, 3.9 km W summit of Mt Ningua, on Boulouparis–Thio Road, about 50 m upstream road, 21°44.359'S, 166°06.009'E, 117 m, 20.xi–12.xii.2003, Malaise trap, loc#035, leg. K.A. Johanson (NHRS).

###### Etymology.

Name *spinifera* is descriptive of the sharp spines of the subgenital processes.

###### Remarks.

This species appears to be quite widespread from the far south towards the north of the island (Fig. 86).

##### Oxyethira (Trichoglene) tiwaka sp. n.

Taxon classificationAnimaliaTrichopteraHydroptilidae

http://zoobank.org/B1B6D5FA-BB85-4891-BE05-7B8F0C950129

Figs 4–6 , 75 , 87

###### Diagnosis.

Readily recognised by the short, blunt, darkly sclerotised peg-like gonopods but in other respects showing very close resemblance to Oxyethira (Trichoglene) spinifera which has gonopods reduced so severely that they can be recognised as only small convexities on the apico-ventral margin of segment IX. Also similar to Oxyethira (Trichoglene) perignonica sp. n. in having abdominal segment IX subquadrate, but that species has the gonopods situated laterally, and curved mesally, and the subgenital processes in the form of convergent, rather than parallel, spines.

###### Description.

Male antennae with 18–19 flagellomeres, flagellomeres all dark, without *sensilla placodea*, each flagellomere about as long as wide; anterior wing length 1.4–1.7 mm (n=8); tibial spurs 0,3,4; abdominal sternite VII with small sharp median spur.

Male, genitalia (Figs 4–6). Abdominal segment IX quadrate in ventral view, slightly concave apico-ventrally, dorsally with proximal margin excised, V-shaped. Gonopods in form of two widely spaced, blunt black pegs. Setose lobes of ventral process angled laterally, ventral processes elongate, widely separated, tapered to narrowly rounded apices. Phallic apparatus elongate, with slender titillator and apically narrow, sinuous spine.

###### Material examined.

**Holotype.** Male, (on slide), New Caledonia, Province Nord, Bouérabate Stream, S Mont Ninndo, along road Barabache–Boulagoma, 20°17.409'S, 164°11.242'E, 60 m, 19.xii.2003–7.i.2004, Malaise trap, loc#089, leg. K.A. Johanson (MNHP).

**Paratypes.** 1 male (on slide), data as for holotype, (NHRS); 1 male, Province Sud., Rivière Bleue, 282 m, stony river, loc 4, 22°05.705'S, 166°38.225'E, Malaise trap, 13–16.xi.2001, leg. K.A. Johanson, T. Pape, B. Viklund (NHRS); 18 males, 11 females (3 on slides), Province Sud, Col d’Amieu, fauna reserve, 415 m, small forest stream, loc 25, 21°33.830'S, 165°45.584'E, Malaise trap, 30.xi–5.xii.2001, leg. K.A. Johanson, T. Pape, B. Viklund (NHRS); 1 male, Province Sud, Monts Kwa Ne Mwa, on road between Noumea and Yaté, Rivière des Pirogues, 22°11.225'S, 166°43.338'E, 100 m, 7.xi.2003, light trap, loc#016, leg. K.A. Johanson (NHRS); 1 male, Province Sud, Mt Dzumac, source stream of Ouinne River, downstream crosspoint to mountain track, 22°01.997'S, 166°28.486'E, 795 m, over about 30 m waterfall, 18.xi–4.xii.2003, Malaise trap, loc#031, leg. K.A. Johanson (NHRS); 2 male, 4 females, Province Sud, W slope Mt Ningua, Kwé Néco Stream, at Camp Jacob, 3.9 km W summit of Mt Ningua, on Boulouparis—Thio Road, about 50 m upstream road, 21°44.083'S, 166°06.298'E, 117 m, 29.xi.2003–12.xii.2003, Malaise trap, loc#053, leg. K.A. Johanson (NHRS); 10 males, Province Sud, W slope Mt Ningua, Kwé Néco, Stream, at Camp Jacob, 3.7 km WNW summit of Mt Ningua, on Boulouparis–Thio Road, about 50 m upstream road, 21°43.613'S, 166°06.567'E, 150 m, 29.xi–12.xii.2003, Malaise trap, loc#054, leg. K.A. Johanson (NHRS); 1 male (on slide), Province Nord, Wemwâdiu stream, 850 m E summit Kögi Mtn, 5 m upstream road, about 200 m S Tiwaka River, 20°49.020'S, 165°14.165'E, 24 m, 6–27.xii.2003, Malaise trap, loc#067, leg. K.A. Johanson (NHRS); 1 male (no genitalia) labelled “sp. D”, Province Sud, Co Rigule Stream, 2.1 km N bridge over Baie de Yaté, 4.3 km S Wé Ngéré, 22°08.147'S, 166° 56.072'E, 14 m, 18.i.2004, light trap, loc#122, leg. K.A. Johanson (NHRS).

###### Etymology.

Named for the river beside which one of the specimens was collected.

###### Remarks.

*Oxyethira tiwaka* was collected quite commonly in the southern region, but at only two disjunct localities in the north (Fig. 87). A photograph of the type locality with the trap is rendered in Fig. 75.

##### Oxyethira (Trichoglene) perignonica sp. n.

Taxon classificationAnimaliaTrichopteraHydroptilidae

http://zoobank.org/7DC78980-D10D-49A9-92AF-E60A7CC8CF7C

Figs 7–9 , 76 , 88

###### Diagnosis.

Male is distinguished from *Oxyethira incurvata* sp. n. which also has the gonopods situated laterally and mesally directed although in *Oxyethira perignonica* they are more spur-like, and the rods of the ventral processes are sharply pointed and convergent.

###### Description.

Male antennae with 17–18 flagellomeres, flagellomeres without *sensilla placodea*, each flagellomere about 1.5 X longer than wide; anterior wing length 1.4–1.7 mm (n=2); tibial spurs 0,2,4; abdominal sternite VII without median spur.

Male, genitalia (Figs 7–9). Abdominal segment IX in ventral view subquadrate, distally truncate with a small tuft of short setae each side of midline. Gonopods in ventral view forming a short, stout, mesally directed spur at each apico-lateral angle; setose lobes and rods of subgenital processes widely separated at bases, rods obliquely arranged, apically convergent; in lateral view, rods sharply down-turned. Phallic apparatus elongate, almost length of segments VII–IX, with a fine titillator and, subapically, a slender spine which in some specimens lies parallel to the length of the phallic apparatus, in others is twisted about it orthogonally.

###### Material examined.

**Holotype.** Male (on slide), New Caledonia, Province Sud, stream draining to Marais de la Rivière Blanche, 5 km SW Pont Pérignon, 22°09.513'S, 166°39.942'E, 180 m, 6–16.xi.2003, Malaise trap, loc#011, leg. K.A. Johanson (MNHP).

**Paratypes.** 2 males (1 on slide), Province Sud, W part of Plaine des lacs, 150 m downstream bridge at La Capture, 22°15.967'S, 166°49.493'E, 261 m, 04–22.xi.2003, Malaise trap, loc#007, leg. K.A. Johanson (NHRS); 1 male, Province Sud, stream draining to Marais de la Rivière Blanche, 1.35 km S Pont Pérignon, 22°08.496'S, 166°42.152'E, 180 m, 6–16.xi.2003, Malaise trap, loc#009, leg. K.A. Johanson (NHRS); 2 males, Province Sud, stream draining to Marais de la Rivière Blanche, 2.25 km SW Pont Pérignon, 22.14158°S, 166.67993°E, 157 m, 6–16.xi.2003, Malaise trap, loc#010, leg. K.A. Johanson (NHRS); 2 males (one headless), same data as for holotype, (NHRS); 1 male (on slide), Province Sud, Monts Kwa Ne Mwa, on road between Noumea and Yaté, 1.5 km E Pic Mouirange, 22°12.545'S, 166°40.246'E, 143 m, 9.xi.2003, light trap, loc#018, leg. K.A. Johanson (NHRS).

###### Etymology.

Named for the bridge on the river near where the holotype was collected.

###### Remarks.

Taken only at several sites in the south of the island (Fig. 88), this species appears to have a highly localised distribution. A photograph of the type locality with the trap is rendered in Fig. 76.

##### Oxyethira (Trichoglene) abbreviata sp. n.

Taxon classificationAnimaliaTrichopteraHydroptilidae

http://zoobank.org/83DD6477-6E0C-40ED-8397-12FA3A34508A

Figs 10–12 , 77 , 89

###### Diagnosis.

Most closely similar to *Oxyethira perignonica* and *Oxyethira tiwaka*, all 3 having males with abdominal segment IX quadrate in ventral view. But *Oxyethira abbreviata* sp. n. is distinguished by having the ventral processes distally rounded and with a short sharp spine angled proximally compared with elongate convergent spines of *Oxyethira perignonica*, and elongate parallel spines of *Oxyethira tiwaka*.

###### Description.

Male antennae with 18–19 flagellomeres, flagellomeres rectangular in profile, *sensilla placodea* absent; anterior wing length 1.4–1.7 mm (n=4); tibial spurs 0,3,4; abdominal sternite VII with small sharp medial spine on distal margin.

Male, genitalia (Figs 10–12). Abdominal segment IX tubular, subquadrate in ventral view, distal margin truncate. Gonopods short, conical, widely separated, ventral processes also very short, rounded distally, in ventral view sharply pointed proximally, no setose lobes apparent. Phallic apparatus elongate, exceeding 3 abdominal segments in length, narrow; slender titillator present and subapically a tightly curved spine.

###### Material examined.

**Holotype.** Male (on slide), New Caledonia, Province Sud, Monts des Koghis, ca 800 m S Koghi Restaurant, 22.18447°S, 166.50315°E, 400 m, 11–26.xi.2003, Malaise trap, loc. 23, leg. K.A. Johanson (MNHP).

**Paratypes.** 2 males, Province Nord, Mt Aoupinié, 354 m, stream, loc. 17, light trap, 24.xi.2001, leg. K.A. Johanson, T. Pape & B. Viklund (NHRS); 1 male, Province Sud, Col d’Amieu, 323 m, small stony river, loc. 24, 21°34.844'S, 165°49.677'E, Malaise trap, 30.xi–5.xii.2001, leg. K.A. Johanson, T. Pape & B. Viklund (NHRS); 1 male, Province Sud, Mt Dzumac, source stream of Ouinne River, at crosspoint to mountain track, 22°02.218'S, 166°28.566'E, 797 m, 18.xi.2003, light trap, loc#032, leg. K.A. Johanson (NHRS); 1 male, Province Sud, Platou de Dogny, source of Dogny River, about 900 m SE summit of Platou de Dogny, 21.61917°S, 165.88072°E, 919 m, 25.xi–16.xii.2003, Malaise trap, loc#046, leg. K.A. Johanson (NHRS).

###### Etymology.

*abbreviata*, named for the very abbreviated male genital structures.

###### Remarks.

This species was collected from several quite central sites (Fig. 89) from small rocky streams. A photograph of the type locality with the trap is rendered in Fig. 77.

##### Oxyethira (Trichoglene) incurvata sp. n.

Taxon classificationAnimaliaTrichopteraHydroptilidae

http://zoobank.org/B6FE045D-C60B-4DCD-AD73-6DDB8921C6D3

**Figures 13–22.**
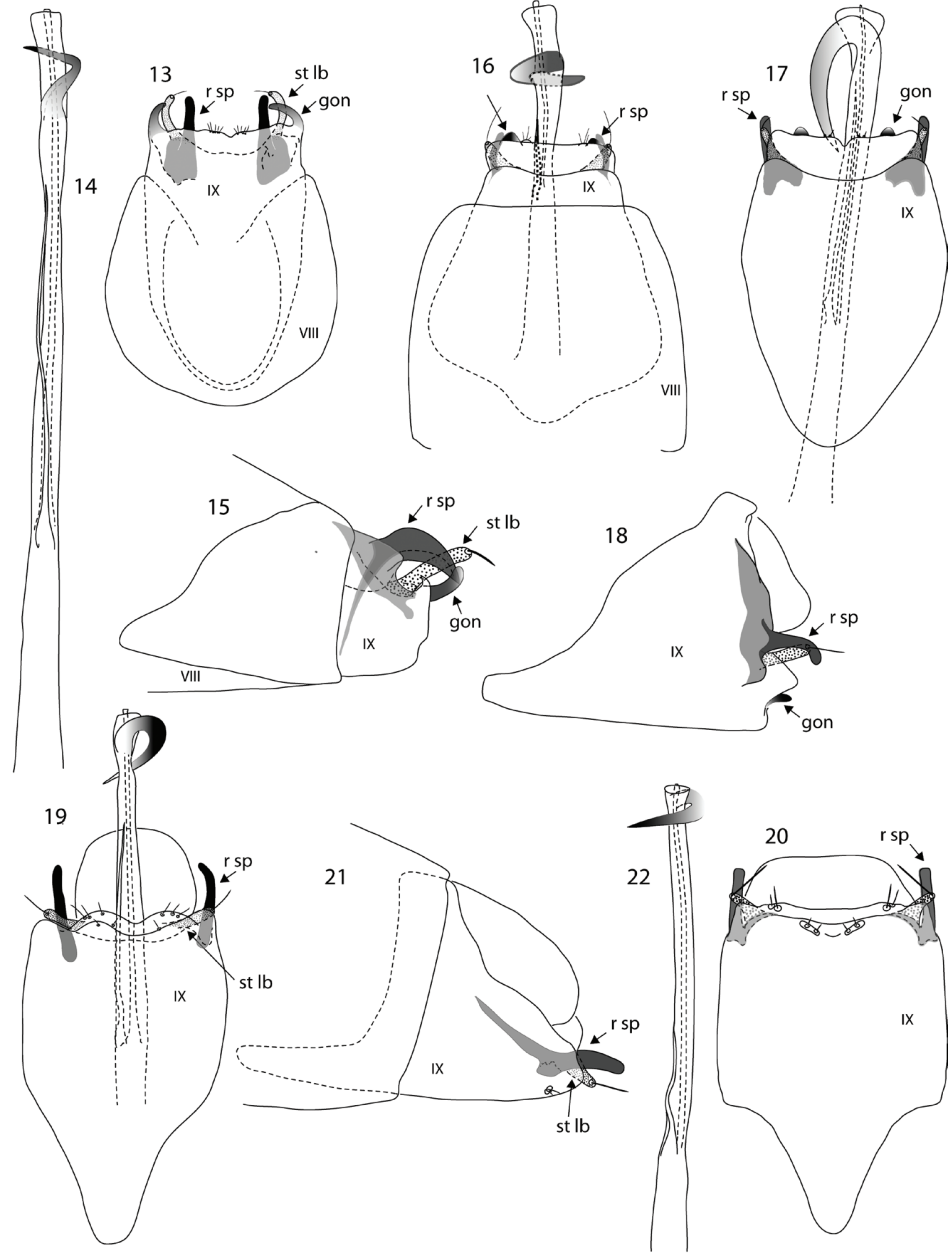
*Oxyethira* species male genitalia. **13–15** *Oxyethira incurvata* sp. n., ventral view, phallic apparatus and lateral view. **16–18** *Oxyethira caledoniensis* Kelley: **16** ventral view of holotype specimen drawn from holotype **17, 18** ventral and later al views of variant form **19–22** *Oxyethira arok* Oláh & Johanson: **19** ventral view drawn from paratype specimen **20–22** ventral and lateral views and phallic apparatus of variant form. Abbreviations: gon = gonopod; r sp = rod of subgenital process; st lb = setose lobe of subgenital process; VIII, IX = abdominal segments VIII and IX.

Figs 13–15 , 78 , 90

###### Diagnosis.

Males resemble superficially those of *Oxyethira perignonica* both having mesally directed, laterally situated gonopods, but in ventral view these are more slender than those of *Oxyethira perignonica* and the rods of the subgenital processes are aligned in parallel with the distal margins of sternite IX in contrast to *Oxyethira perignonica* in which the they form sharp spines angled obliquely.

###### Description.

Male antennae with 20–25 flagellomeres, flagellomeres with few *sensilla placodea*, each rectangular in profile and 1.5–2× longer than wide; anterior wing length 1.2–1.5 mm (n=8); tibial spurs 0,3,4; abdominal sternite VII without median spur.

Male, genitalia (Figs 13–15). Abdominal segments VIII and IX rounded proximally. Abdominal sternite IX truncate distally, small areas of short setae apically each side of midline. Gonopods forming two strongly in-curved sclerotised processes at apico-lateral angles, setose lobes and sclerotised rods of subgenital processes widely separated, rods tapered to narrowly rounded apices. Phallic apparatus elongate, a slender sinuous spine subapically.

###### Material examined.

**Holotype.** Male, New Caledonia, Province Nord, Mt Panié, 20.57306°S, 164.77139°E, 902 m, 9.xii.2003, Malaise trap, loc#075, leg. K.A. Johanson (MNHP).

**Paratypes.** 4 males (2 on slides), Province Sud., Rivière Bleue, 282 m, stony river, loc 4, 22°05.705'S, 166°38.225'E, Malaise trap, 13–16.xi.2001, leg. K.A. Johanson, T. Pape & B. Viklund (NHRS); 5 males, Province Sud, stream draining to Marais de la Rivière Blanche, 1.35 km S Pont Pérignon, 22°08.496'S, 166°42.152'E, 180 m, 6–16.xi.2003, Malaise trap, loc#009, leg. K.A. Johanson (NHRS); 5 males, Province Sud, side stream to Rivière Blanche, 10.75 km SW Pont Pérignon, 22°10.073'S, 166°39.903'E, 180 m, 6–16.xi.2003, Malaise trap, loc#012, leg. K.A. Johanson (NHRS); 1 male, Province Sud, Mt Dzumac, source stream of Ouinne River, near crosspoint to mountain track, 22°02.073'S, 166°28.460'E, 810 m, 18.xi–4.xii.2003, Malaise trap, loc#030, leg. K.A. Johanson (NHRS); 3 males (1 on slide), Province Sud, Mt Dzumac, source stream of Ouinne River, downstream crosspoint to mountain track, 22°01.997'S, 166°28.486'E, 795 m, over about 30 m waterfall, 18.xi–4.xii.2003, Malaise trap, loc#031, leg. K.A. Johanson (NHRS); 1 male, Province Sud, Mt Dzumac, source stream of Ouinne River, near crosspoint to mountain track, 22°02.439'S, 166°28.646'E, 805 m, 18.xi–4.xii.2003, Malaise trap, loc#029, leg. K.A. Johanson (NHRS).

###### Etymology.

The name *incurvata* is descriptive of the orientation of the gonopods.

###### Remarks.

From the collecting records the distribution of this species appears to be highly disjunct (Fig. 90), with records from the northern and southern extremes of the island. A photograph of the type locality with the trap is rendered in Fig. 78.

##### Oxyethira (Trichoglene) caledoniensis

Taxon classificationAnimaliaTrichopteraHydroptilidae

Kelley

Figs 16–18 , 75 , 91

Oxyethira caledoniensis Kelley, 1989: 196, figs 33, 42, 56.

###### Revised diagnosis.

A typical member of subgenus *Trichoglene*, with males superficially difficult to distinguish from other closely similar species such as *Oxyethira abbreviata*, *Oxyethira arok* and *Oxyethira spinifera*. Neither *Oxyethira arok* nor *Oxyethira spinifera* sp. n. has recognisable gonopods whereas both *Oxyethira caledoniensis* and *Oxyethira abbreviata* sp. n. have widely separated, shallowly dome-shaped gonopods; *Oxyethira abbreviata* sp. n. has very short, sharply pointed subgenital processes that are directed anteriorly and abdominal segment IX quadrate in ventral view, compared with the rod-shaped dorsal processes of *Oxyethira caledoniensis* and sub-triangular to shield-shaped abdominal segment IX.

Antennae: male with 19–24 flagellomeres, all dark; flagellomeres rectangular in profile, without *sensilla placodea*; female with 15 flagellomeres. Anterior wing length: male 1.4–1.9 mm (n=12); female 1.4–1.7 mm (n= 8). Tibial spurs 0,3,4. Abdominal segment VII with mid-ventral sharp spine.

###### Material examined.

**Holotype.** Male, New Caledonia, Plum, (BPBM). **Other material.** 1 male, province Sud, Ouenghi River, Boulouparis, 19.xii.1983, AW (ANIC); 4 males, Province Sud., Rivière Bleue, 282 m, stony river, loc 4, 22°05.705'S, 166°38.225'E, Malaise trap, 13–16.xi.2001, leg. K.A. Johanson, T. Pape & B. Viklund(NHRS); 4 males (on slides), Province Sud, stream draining to Marais de la Rivière Blanche, 1.35 km S Pont Pérignon, 22°08.496'S, 166°42.152'E, 180 m, 6–16.xi.2003, Malaise trap, loc#009, leg. K.A. Johanson (NHRS); 2 males, Province Sud, stream draining to Marais de la Rivière Blanche, 2.25 km SW Pont Pérignon, 22.14158°S, 166.67993°E, 157 m, 6–16.xi.2003, Malaise trap, loc#010, leg. K.A. Johanson (NHRS); 3 males (on slides), females, Province Sud, Mt Dzumac, source stream of Ouinne River, near crosspoint to mountain track, 22°02.439'S, 166°28.646'E, 805 m, 18.xi–4.xii.2003, Malaise trap, loc#029, leg. K.A. Johanson (NHRS); 9 males, Province Sud, Mt Dzumac, source stream of Ouinne River, near crosspoint to mountain track, 22°02.073'S, 166°28.460'E, 810 m, 18.xi–4.xii.2003, Malaise trap, loc#030, leg. K.A. Johanson (NHRS); 9 males, 8 females, Province Sud, Mt Dzumac, source stream of Ouinne River, downstream crosspoint to mountain track, 22°01.997'S, 166°28.486'E, 795 m, over about 30 m waterfall, 18.xi–4.xii.2003, Malaise trap, loc#031, leg. K.A. Johanson (NHRS); 2 males, Province Sud, W slope Mt Ningua, Kwé Néco Stream, 3.9 km W summit of Mt Ningua, on Boulouparis–Thio Road, about 50 m upstream road, 21°44.359'S, 166°06.009'E, 117 m, 20.xi–12.xii.2003, Malaise trap, loc#035leg. K.A. Johanson (MNHN); 1 male, Province Sud, on road between Noumea and Yaté, 1.0 km NW Pont des Japonais, 22°11.427'S, 166°42.868'E, 113 m, 22.xi–4.xii.2003, Malaise trap, loc#039, leg. K.A. Johanson (NHRS);10 males (5 on slides), Province Sud, W slope Mt Ningua, Kwé Néco, Stream, at Camp Jacob, 3.7 km WNW summit of Mt Ningua, on Boulouparis–Thio Road, about 50 m upstream road, 21°43.613'S, 166°06.567'E, 150 m, 29.xi–12.xii.2003, Malaise trap, loc#054, leg. K.A. Johanson (NHRS, ANIC); 59 males, 15 females, Province Nord, Mt Panié, stream at camp, 20.58167°S, 164.76472°E, 1311 m, 9.xii.2003, Malaise trap, loc#073, leg. K.A. Johanson (NHRS); 1 male, Province Nord, Mt Panié, stream at camp, 20. 58139°S, 164.76444°E, 1310 m, 9.xii.2003–2.i.2004, Malaise trap, loc#074, leg. K.A. Johanson (NHRS); 23 males, 3 females, Province Nord, Mt Panié, 20.57306°S, 164.77139°E, 902 m, 9.xii.2003, Malaise trap, loc#075, leg. K.A. Johanson (NHRS); 3 males, Province Nord, stream in Creek de Bambou, 5 m N road RT7 Ouégoa–Koumac, 20°27.863'S, 164°19.784'E, 58 m, 19.xii.2003, Malaise trap, loc#087, leg. K.A. Johanson (NHRS); 4 males (2 on slides), Province Nord, Bouérabate Stream, S Mont Ninndo, along road Barabache–Boulagoma, 20°17.409'S, 164°11.242'E, 60 m, 19.xii.2003–7.i.2004, Malaise trap, loc#089, leg. K.A. Johanson (NHRS); 5 males, Province Nord, Forêt Plate, Ouendé River, at 2.5 km WNW summit of Katépouenda, 23.3 km E Pouembout, 21°07.490'S, 165°06.723'E, 470 m, 8–15.i.2004, Malaise trap, loc#112, leg. K.A. Johanson (NHRS).

###### Remarks.

Delineation of this species among the large collection before us proved difficult, with only a very few specimens conforming closely to the holotype. In describing the species, Kelley (1989) had access to only a single specimen, re-examination of which shows it to be as illustrated in Kelley’s fig. 56 (redrawn here from the type in Fig. 14), except that the subgenital processes are gently curved mesally, not slightly sinuous as figured by Kelley. A considerable number of specimens have been examined that agree in general features, but have abdominal segment IX either shorter but much the same shape as in the holotype male, or more elongate and rounded anteriorly; in some the spine on the phallic apparatus is longer and more strongly recurved and arising closer to the apex than in the type. This latter form is illustrated in Fig. 17 and was initially thought to be a separate species. However, following examination of further material of forms intermediate between *Oxyethira caledoniensis* sensu Kelley and this particular form, it is included tentatively as a variant form of *Oxyethira caledoniensis* together with all newly available specimens with the apico-ventral margin of abdominal segment IX truncate, sometimes with some slight marginal sclerotisation, the gonopods reduced to short domes, and abdominal segment IX sub-triangular to conical. Future studies may show reveal that these represent more than one species. *Oxyethira caledoniensis* is recorded from sites along the length of the island, but most commonly in the far south (Fig. 91). A photograph of one of the northern collecting sites is shown in Fig. 75, the type locality of *Oxyethira tiwaka* and *Oxyethira ouenghi*, and shared with a number of other *Oxyethira* species.

##### Oxyethira (Trichoglene) arok

Taxon classificationAnimaliaTrichopteraHydroptilidae

Oláh & Johanson

Figs 19–22 , 92

Oxyethira arok Oláh & Johanson, 2010a: 91, figs 42–44.

###### Revised diagnosis.

In general appearance of male genitalia *Oxyethira arok* is closest to *Oxyethira caledoniensis* but appears to have gonopods reduced completely, which feature it shares with *Oxyethira amieu* sp. n. and *Oxyethira spinifera*; however, *Oxyethira arok* has abdominal segment IX almost parallel-sided in distal half, rather than tapered as in *Oxyethira amieu* sp. n., and *Oxyethira spinifera* has the rods of subgenital processes tapered to acute apices compared with the blunt apices of *Oxyethira arok*.

Antennae: male with 20–21 flagellomeres; flagellomeres quadrate to slightly rectangular in profile; female with 17 flagellomeres, terminal 2 pale, rest dark. Anterior wing length: male 1.2–1.8 mm (n=10); female 1.4–1.6 mm (n =10). Tibial spurs 0,3,4. Abdominal sternite VII with small median spur offset from distal margin. Female with length of abdominal segment IX almost twice width, distal margin, with a sclerotised margin, mesally produced distally. Segment X stouter at base than apex, gradually tapered distally, truncate apically.

###### Material examined.

**Paratype.** 1 male, New Caledonia, Province Sud, Monts Kwa Ne Mwa, on road between Nouméa andYaté, 2.0 km E Pic Mouirange, 22°12.356'S, 166°40.798'E, 220 m, 16–30.xi.2003, Malaise trap, loc#014, leg. K.A. Johanson. **Other material.** 5 males, Province Nord, Mt Aoupinié, 354 m, stream, loc 17, light trap, 24.xi.2001, leg. K.A. Johanson, T. Pape & B. Viklund (NHRS); 2 males, Province Sud, Monts des Koghis, ca 300 m S Koghi Restaurant, 22.18288°S, 166.50167°E, 417 m, 2–16.xi.2003, Malaise trap, loc#004, leg. K.A. Johanson (NHRS); 11 males, Province Sud, Monts Kwa Ne Mwa, on road between Noumea and Yaté, Rivière des Pirogues, 22°11.225'S, 166°43.338'E, 100 m, 7.xi.2003, light trap, loc#016, leg. K.A. Johanson (NHRS); numerous males (5 on slides), females (5 on slides), Province Sud, Monts Kwa Ne Mwa, on road between Nouméa and Yaté, 2.0 km E Pic Mouirange, 22°12.356'S, 166°40.798'E, 220 m, 7–16.xi.2003, Malaise trap, loc#014, leg. K.A. Johanson (NHRS); 2 males, Province Nord, Aoupinié Mtn, Réserve spéciale de faune de l’Aoupinié, spring to side stream to Öröpömwati river, 21°09.032'S, 165°19.179'E, 441 m, 6–27.xii.2003, Malaise trap, loc#065, leg. K.A. Johanson (NHRS).

###### Remarks.

Specimens here identified as *Oxyethira arok* show some variability in proportions of abdominal segment IX and in male genital structures as apparent in Figures 17–19, but for the present these differences are considered insignificant.

*Oxyethira arok* has been collected from disjunct localities in the far south and central part of the island (Fig. 92).

##### Oxyethira (Trichoglene) amieu sp. n.

Taxon classificationAnimaliaTrichopteraHydroptilidae

http://zoobank.org/B4BFF0C3-89A6-4825-A833-EFDFB92E5BD8

Figs 23 , 24 , 93

###### Diagnosis.

Males are similar to *Oxyethira arok* and *Oxyethira houailou* sp. n. in the shape of abdominal segment IX, which in ventral view is strongly tapered and more or less triangular proximally, but both *Oxyethira amieu* sp. n. and *Oxyethira houailou* sp. n. also taper distally, while *Oxyethira arok* is more or less parallel-sided in distal half; in *Oxyethira amieu* sp. n. and *Oxyethira arok* gonopods are so reduced they cannot be identified clearly whereas in ventral view they are subquadrate in *Oxyethira houailou* sp. n.

###### Description.

Male antennae with 22 flagellomeres; flagellomeres rectangular in profile, without *sensilla placodea*; anterior wing length 1.4 mm (n=1); tibial spurs 0,3,4; abdominal sternite VII with a short slender apico-mesal spur.

**Figures 23–34.**
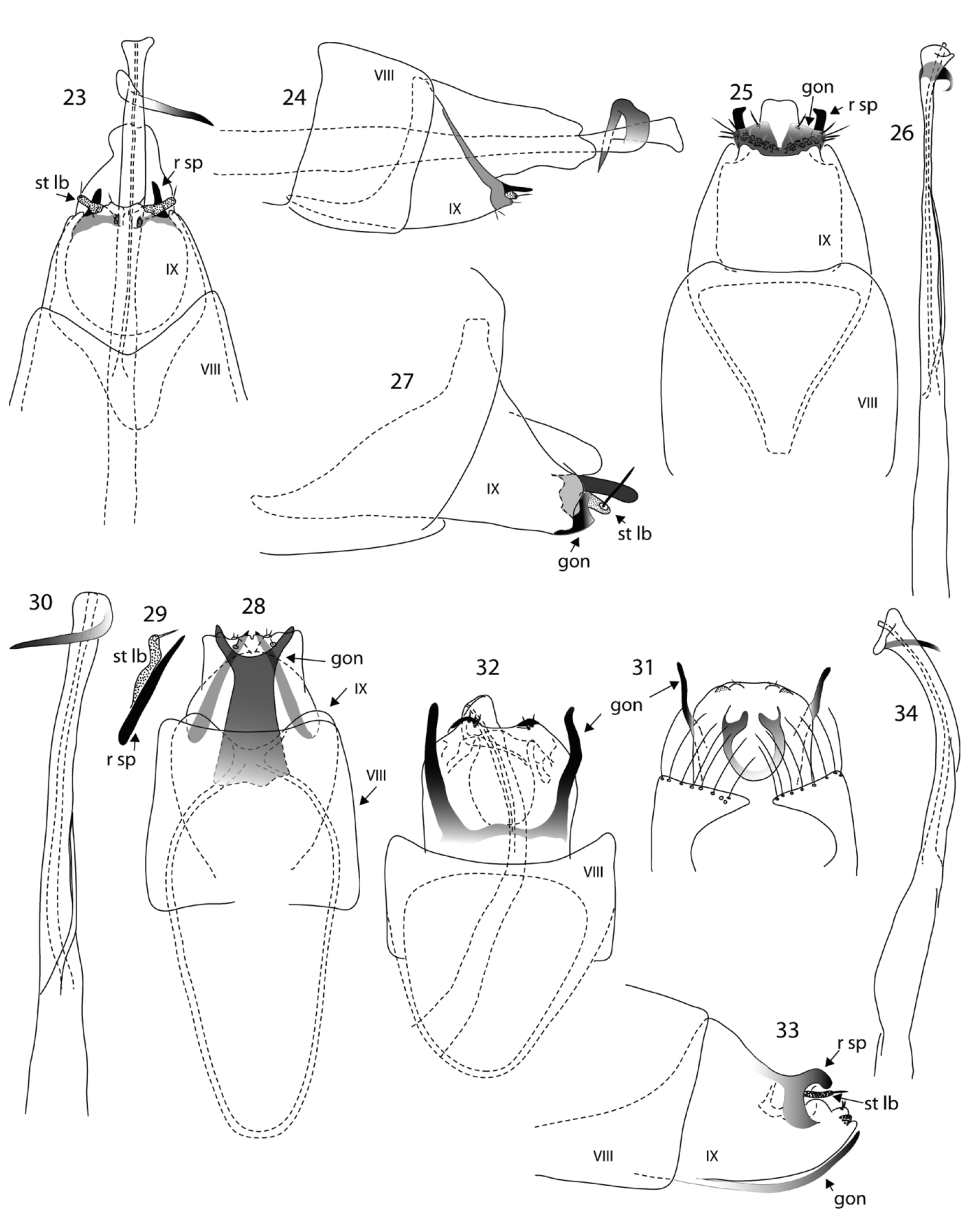
*Oxyethira* species male genitalia. **23, 24** *Oxyethira amieu* sp. n., ventral and lateral views **25–27** *Oxyethira houailu* sp. n., ventral view, phallic apparatus and lateral view **28–30** *Oxyethira insularis* Kelley, ventral view, spine and setose lobe of left subgenital process and phallic apparatus **31–34** *Oxyethira parinsularis* sp. n., dorsal, ventral and lateral views and phallic apparatus. Abbreviations: gon = gonopod; r sp = rod of subgenital process; st lb = setose lobe of subgenital process; VIII, IX = abdominal segments VIII and IX.

Male, genitalia (Figs 23, 24). Abdominal segment IX in ventral view subtriangular in proximal half, with proximal margin broadly rounded, distally tapered to about half maximum width; in lateral view triangular; gonopods reduced completely, subgenital processes rod-like, tapered distally, setal lobes almost at right angles to length of body; phallic apparatus with slender titillator and narrow, elongate subapical spine.

###### Material examined.

**Holotype.** Male, New Caledonia, Chute, ~15 km N Col d’Amieu on La Foa–Canala Rd, xii.1998, A. Wells, (MNHP).

###### Etymology.

Named for the Col d’Amieu.

###### Remarks.

Known only from the type locality, a waterfall towards the top of the massif (Fig. 93).

##### Oxyethira (Trichoglene) houailou sp. n.

Taxon classificationAnimaliaTrichopteraHydroptilidae

http://zoobank.org/5EDFA209-521B-4CE3-83FA-7C99B781B4C7

Figs 25–27 , 94

###### Diagnosis.

Males of *Oxyethira houailou* sp. n. resemble those of *Oxyethira amieu* and *Oxyethira arok*, but are distinguished by having gonopods quadrate in ventral view and separated by mid-ventral V-shaped excision, by having mesally directed apices on ventral processes, and spine on the phallic apparatus subapical and short compared with longer spines of the other two species

###### Description.

Male antennae with 21–27 flagellomeres; flagellomeres subquadrate in profile, without *sensilla placodea*, but with dense *sensilla auricillica*; anterior wing length, male 1.4–1.9 mm (n=10); tibial spurs 0,3,4; abdominal sternite VII with a short sharp mesal spur.

Male, genitalia (Figs 25–27). Abdominal segment IX in ventral view subtriangular in proximal half, with proximal margin truncate to broadly rounded, distally tapered to about half maximum width at distal margin; in lateral view triangular. Subgenital processes in form of stout sclerotised rods, their apices blunt, and short setose lobes, in ventral view almost at right angles to length of body. Phallic apparatus elongate, slender, with fine titillator, and subapically a short curved spine. Abdominal tergite X only slightly longer than rods of ventral processes, membranous.

Female unknown.

###### Material examined.

**Holotype.** Male (on slide), New Caledonia, Province Nord, small fall ~10 km SW Houaïlou, on Bourail road, 16.xii.1998, A. Wells (MNHP).

**Paratypes.** New Caledonia: 1 male (on slide), data as for holotype, (ANIC); 1 male, Province Sud, Col d’Amieu, 323 m, small stony river, loc 24, 21°34.844'S, 165°49.677'E, Malaise trap, 30.xi–5.xii.2001, leg. K.A. Johanson, T. Pape & B. Viklund (NHRS); 6 males, Province Sud, Monts Kwa Ne Mwa, on road between Noumea and Yaté, Rivière des Pirogues, 22°11.225'S, 166°43.338'E, 100 m, 7.xi.2003, light trap, loc#016, leg. K.A. Johanson (NHRS); 1 male, Province Sud, Mt Dzumac, source stream of Ouinne River, near crosspoint to mountain track, 22°02.439'S, 166°28.646'E, 805 m, 18.xi–4.xii.2003, Malaise trap, loc#029, leg. K.A. Johanson (NHRS); 2 males, Province Sud, Mt Dzumac, source stream of Ouinne River, near crosspoint to mountain track, 22°02.073'S, 166°28.460'E, 810 m, 18.xi–4.xii.2003, Malaise trap, loc#030, leg. K.A. Johanson (NHRS); 1 male, Province Sud, Platou de Dogny, source of Dogny River, about 1.2km SE summit of Platou, about 200 m from waterfall, 21.62067°S, 165.88290°E, 915 m, 25.xi–16.xii.2003, Malaise trap, loc#048, leg. K.A. Johanson (NHRS); 1 male (on slide), Province Nord, Wé Caot Stream, draining NNE side of Mt Panié, 0.9 km NW Cascade de Tao, 20°33.311'S, 164°48.064'E, 18.xii.2003, light trap, loc#084, leg. K.A. Johanson (NHRS).

###### Etymology.

Named for the settlement of Houaïlou, near the type locality.

###### Remarks.

This species was collected from widespread localities along the length of the island (Fig. 94).

##### Oxyethira (Trichoglene) insularis

Taxon classificationAnimaliaTrichopteraHydroptilidae

Kelley

Figs 28–30 , 95

Oxyethira insularis Kelley, 1989: 196, figs 34, 43, 57.

###### Revised diagnosis.

This species shares with *Oxyethira parinsularis* sp. n. the feature of gonopods, in ventral view, in the form of a prominent, sclerotised, Y-shaped structure, arising at around the distal third of abdominal segment IX, but differs from *Oxyethira parinsularis* sp. n. by having the fused basal section over 2× length of the divergent distal arms, not forming a shallow sclerotised band, and the bifid distal arms about 1/3 length of basal stem, not greatly exceeding length of base as in *Oxyethira parinsularis* sp. n.

Male antennae with 18 flagellomeres; flagellomeres rectangular in profile, without *sensilla placodea*, with abundant *sensilla auricillica*. Anterior wing length 1.5–1.8 mm (n=3). Tibial spurs 0,3,4. Venter of abdominal sternite VII with median spine.

###### Material examined.

**Holotype.** Male, New Caledonia, mountain stream up Boulari River, (BPBM). **Other material.** 2 males (1 on slide), Province Sud, Mt Dzumac, source stream of Ouinne River, near crosspoint to mountain track, 22°02.073'S, 166°28.460'E, 810 m, 18.xi–4.xii.2003, Malaise trap, loc#030, leg. K.A. Johanson (NHRS); 1 male, Province Sud, on road between Noumea and Yaté, 1.0 km NW Pont des Japonais, 22°11.427'S, 166°42.868'E, 113 m, 22.xi–4.xii.2003, Malaise trap, loc#039, leg. K.A. Johanson.

###### Remarks.

Among the extensive collection of New Caledonian *Oxyethira* at hand, only three specimens of this species were identified, all from the south of the island (Fig. 95). Other specimens from one of the sites at which they were taken are distinct and are referred to *Oxyethira parinsularis* sp. n.

##### Oxyethira (Trichoglene) parinsularis sp. n.

Taxon classificationAnimaliaTrichopteraHydroptilidae

http://zoobank.org/FCEEFD2A-73D2-4DC0-BADF-FA58E6EA630B

Figs 31–34 , 79 , 96

###### Diagnosis.

Males are distinguished from those of the closely similar *Oxyethira insularis* by the shape of gonopods which have longer, more elongate and slender divergent arms and very short, fused basal portion.

###### Description.

Male antennae with 18–19 flagellomeres, flagellomeres rectangular in profile, without *sensilla placodea*, with numerous *sensilla auricillica*; anterior wing length 1.5–1.8 mm (n=7); tibial spurs 0,3,4; abdominal sternite VII with median spine.

Male, genitalia (Figs 31–34). Abdominal segment IX rounded proximally, in ventral view apically almost truncate but shallowly excavated medially, in dorsal view deeply and roundly excavated, with a pair of short, curved sclerotised processes mesally, interpreted as homologues of ventral processes, with associated short setose lobes; gonopods forked, forming a pair of widely divergent slender, curved spines, basally fused in a narrow band; phallic apparatus with a strongly recurved narrow spine apically; titillator present.

###### Material examined.

**Holotype.** Male (on slide), New Caledonia, Province Sud, Mt Dzumac, source stream of Ouinne River, near crosspoint to mountain track, 22°02.073'S, 166°28.460'E, 810 m, 18.xi–4.xii.2003, Malaise trap, loc#030, leg. K.A. Johanson, (MNHP).

**Paratypes.** 12 males (2 on slides), data as for holotype, (NHRS); 8 males, Province Sud, Mt Dzumac, source stream of Ouinne River, near crosspoint to mountain track, 22°02.439'S, 166°28.646'E, 805 m, 18.xi–4.xii.2003, Malaise trap, loc#029, leg. K.A. Johanson (NHRS); 1 male, New Caledonia, Province Sud, Plateau de Dogny, source of Dogny River, about 1.4 km SE summit of Plateau, about 20 m upstream waterfall, 21.62054°S, 165.88503°E, 912 m, 25.xi–16.xii.2003, Malaise trap, loc#049, leg. K.A. Johanson, leg. K.A. Johanson (NRMS); 1 male (on slide), Province Sud, W slope Mt Ningua, Kwé Néco, Stream, at Camp Jacob, 3.7 km WNW summit of Mt Ningua, on Boulouparis–Thio Road, about 50 m upstream road, 21°43.613'S, 166°06.567'E, 150 m, 29.xi–12.xii.2003, Malaise trap, loc#054, leg. K.A. Johanson (ANIC).

###### Remarks.

The close similarity between this species and *Oxyethira insularis* is worrying, especially since both were taken at one site, yet the differences are clear and consistent. The species was taken only in the southern province (Fig. 96). A photograph of the type locality with the trap is rendered in Fig. 79.

#### Subgenus *Pacificotrichia* Kelley, 1989

Subgenus *Pacificotrichia*, based on the type species, *Oxyethira oropedion* Kelley, was diagnosed by Kelley (1989) by the following features of males: “… shallowly excised venter VIII …, the deeply excised dorsum VIII …, fused R4 and R5 forewing veins, configuration of the subgenital processes [these are fused], and reduction or loss of the pre-apical spur on the meso-tibia”. Kelley commented on the similarity between the genitalia of males of this subgenus and those of the *minima* group in subgenus *Dampfitrichia*, but noted that in *Pacificotrichia* the subgenital processes are “distally fused … do not bear distal setae … and retain the bilobed process”. The structure formed by the fused subgenital processes is generally characteristic for species, forming, in ventral view, what appears to be a plate ventral to the phallic apparatus. In common with *minima* group species in *Dampfitrichia*, most species in the *Pacificotrichia* group have a slender mid-ventral apodeme usually almost as long as the venter of abdominal segment IX, and unlike members of subgenus *Trichoglene*, lack a titillator on the phallic apparatus.

Seven species were referred by Kelley (1989) to subgenus *Pacificotrichia*, among which five were referred to an *oropedion* group and two to an *efatensis* group; the latter group was recorded only from Fiji and Vanuatu (one species each). However he did not define the two groups, and retention of these groups seems unnecessary.

The following New Caledonian species are referred to this subgenus: *Oxyethira oropedion* (including *Oxyethira derek* Oláh & Johanson (syn. n.)); *Oxyethira quadrata* sp. n.; *Oxyethira dorsennus* Kelley; *Oxyethira rougensis* sp. n.; *Oxyethira indorsennus* Kelley (including *Oxyethira tompa* Oláh & Johanson (**syn. n.**); *Oxyethira melasma* Kelley; *Oxyethira nehoue* sp. n.; *Oxyethira ouenghi* sp. n.; *Oxyethira mouirange* sp. n.; *Oxyethira enigmatica* sp. n.; *Oxyethira scutica* Kelley, *Oxyethira spicula* sp. n. and *Oxyethira digitata* sp. n.

##### Oxyethira (Pacificotrichia) oropedion

Taxon classificationAnimaliaTrichopteraHydroptilidae

Kelley

**Figures 35–43.**
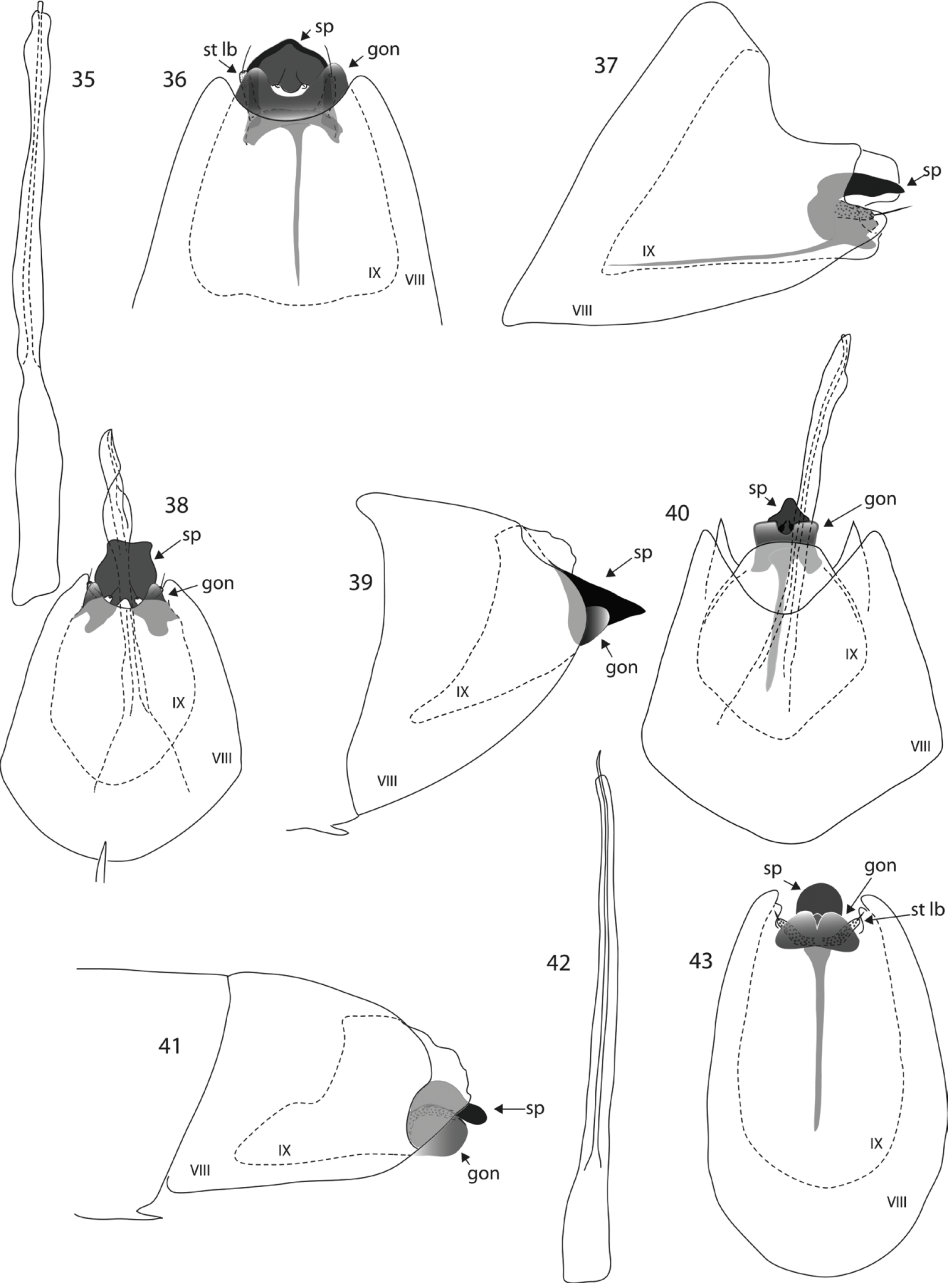
*Oxyethira* species male genitalia. **35–37** *Oxyethira oropedion* Kelley, phallic apparatus, ventral and lateral views **38** *Oxyethira quadrata* sp. n. ventral view **39, 40** *Oxyethira dorsennus* Kelley, lateral and ventral views **41–43** *Oxyethira indorsennus* Kelley, lateral view, phallic apparatus and ventral view. Abbreviations: gon = gonopod; sp = subgenital process; st lb = setose lobe of subgenital process; VIII, IX = abdominal segments VIII and IX.

Figs 35–37 , 78 , 79 , 97

Oxyethira oropedion Kelley, 1989: 198, figs 38, 46, 50, 51, 60.
Oxyethira derek Oláh & Johanson, 2010a: 95, figs 49–51. **syn. n.**

###### Revised diagnosis.

Males are readily recognised by the short, setose mid-apicoventral lobes situated between the gonopods [present in the holotype, although not illustrated clearly by Kelley (1989: fig. 60)]. In other respects they resemble *Oxyethira quadrata* sp. n., *Oxyethira indorsennus*, and *Oxyethira rougensis* sp. n. but differ in ventral view from *Oxyethira quadrata* sp. n. in the apically subtriangular to rounded shape of the subgenital process, rather than quadrate, and the simple, straight phallic apparatus lacking distal flanges; from *Oxyethira indorsennus* in the wider separation of the gonopods; and from *Oxyethira rougensis* sp. n. by the broad, shallow excision of abdominal segment VIII, rather than deep almost V-shaped excision. The female is distinctive in having abdominal segments IX and X slender with a small jet black spot ventrally on abdominal segment X (Kelley 1989: figs 50, 51 unlike the female of *Oxyethira incana* Ulmer which has the entire ventral surface of abdominal segment X very darkly sclerotised.

Antennae: male 22–23 flagellomeres, banded with terminal 3 flagellomeres pale, then 5 dark, 1 light, 2 dark 4 light, then dark to base, flagellomeres bearing *sensilla placodea*; female antennae with 19 flagellomeres, with terminal 3 flagellomeres pale, 5 dark, 5 light, then dark to base. Wing length male 1.6–1.9 mm; female 1.4–1.9 mm. Tibial spurs 0,3,4. Abdominal sternite VII with median spur on distal margin.

###### Material examined.

**Holotype.** Male, New Caledonia, Plateau de Dogny, (BPBM). **Other material.** 1 males (on slide), Province Sud, NW of Farino on Moindou road, ~10 km, ford at confluence, 20.xii.1998, A. Wells (ANIC); 1 male (on slide) small fall ~20 km SW Houailou, on Houailou–Bourail road, 26.xii.1998, AW (ANIC); 1 male (on slide), chute ~15km N Col d’Amieu on La Foa–Canala Rd], 27.XII. 1998 AW (ANIC); 1 male (on slide), La Foa, 27.xii.1998, A Wells, (ANIC); 1 male (on slide), stream ~15 km SW Thio on Boulouparis–Thio road, 28.xii.1998, AW (ANIC); 4 males, Province Sud., Rivière Bleue, 282 m, stony river, loc 4, 22°05.705'S, 166°38.225'E, Malaise trap, 13–16.xi.2001, leg. K.A. Johanson, T. Pape & B. Viklund(NHRS); 3 males, 7 females, Province Sud, Sarraméa, 220 m, forest stream, loc 10, 21°37.883'S 165°51.958'E, Malaise trap, 18–21.xi.2001, leg. K.A. Johanson, T. Pape & B. Viklund (NHRS); 6 males, 26 females, Province Sud, Sarraméa, 2907 m, stony forest stream, loc 13, 21°37.097'S 165°49.351'E, Malaise trap, 18–21.xi.2001, leg. K.A. Johanson, T. Pape & B. Viklund(NHRS); numerous males, females, Province Nord, Amoa River, 23 m, loc 20, 12 km W Poindimié, 22°58.092'S, 165°11.804'E, light trap, 26.xi.2001, Leg. K.A. Johanson, T. Pape & B. Viklund leg. K.A. Johanson, T. Pape & B. Viklund(NHRS); 4 males, 7 females, Province Sud, Col d’Amieu, 323 m, small stony river, loc 24, 21°34.844'S, 165°49.677'E, Malaise trap, 30.xi–5.xii.2001, leg. K.A. Johanson, T. Pape & B. Viklund(NHRS); 27 males, Province Sud, stream draining to Marais de la Rivière Blanche, 5.0 km SW Pont Pérignon, 22°09.513'S, 166°39.942'E, 180 m, 6–16.xi.2003, Malaise trap, loc#011, leg. K.A. Johanson (NHRS); 2 males, Province Sud, Monts Kwa Ne Mwa, on road between Noumea and Yaté, Rivière des Pirogues, 22°11.225'S, 166°43.338'E, 100 m, 7.xi.2003, light trap, loc#016, leg. K.A. Johanson (NHRS); 3 males, 2 females, Province Sud, Mt Dzumac, source stream of Ouinne River, near crosspoint to mountain track, 22°02.439'S, 166°28.646'E, 805 m, 18.xi–4.xii.2003, Malaise trap, loc#029, leg. K.A. Johanson (NHRS); numerous males, females, Province Sud, Mt Dzumac, source stream of Ouinne River, near crosspoint to mountain track, 22°02.073'S, 166°28.460'E, 810 m, 18.xi–4.xii.2003, Malaise trap, loc#030, leg. K.A. Johanson (NHRS); numerous males, females, Province Sud, Mt Dzumac, source stream of Ouinne River, near crosspoint to mountain track, 22°02.439'S, 166°28.646'E, 805 m, 18.xi–4.xii.2003, Malaise trap, loc#029, leg. K.A. Johanson (NHRS); numerous males, females, Province Sud, Tamoa River, 700m S road RT1 between Noumea and La Foa, 22°04.518'S, 166°16.592'E, 19.xi.2003, light trap, loc#033, leg. K.A. Johanson (NHRS); 3 males, 4 females, Province Sud, Hwa Hace Mtn, Hwa Motu River, at Pont Wamuttu, 1.0 km E Nassirah, about 200 m upstream bridge, 21°48.094'S, 166°04.298'E, 137 m, 20.xi–12.xii.2003, Malaise trap, loc#034, leg. K.A. Johanson (NHRS); numerous males, Province Sud, W slope Mt Ningua, Kwé Néco Stream, 3.9 km W summit of Mt Ningua, on Boulouparis–Thio Road, about 50 m upstream road, 21°44.359'S, 166°06.009'E, 117 m, 20.xi–12.xii.2003, Malaise trap, loc#035, leg. K.A. Johanson (NHRS); 2 male, 3 females, Province Sud, on road between Noumea and Yaté, 1.0 km NW Pont des Japonais, 22°11.427'S, 166°42.868'E, 113 m, 22.xi–4.xii.2003, Malaise trap, loc#039, leg. K.A. Johanson (NHRS); numerous males (1 on slide), females 1 on slide), Province Sud, Platou de Dogny, source of Dogny River, about 1.2km SE summit of Platou, about 200 m from waterfall, 21.62067°S, 165.88290°E, 915 m, 25.xi–16.xii.2003, Malaise trap, loc#048, leg. K.A. Johanson (NHRS); numerous males, females, Province Sud, W slope Mt Ningua, Kwé Néco Stream, at Camp Jacob, 3.9 km W summit of Mt Ningua, on Boulouparis—Thio Road, about 50 m upstream road, 21°44.083'S, 166°06.298'E, 117 m, 29.xi.2003–12.xii.2003, Malaise trap, loc#053, leg. K.A. Johanson (NHRS); 17 males Province Sud, W slope Mt Ningua, Kwé Néco, Stream, at Camp Jacob, 3.7 km WNW summit of Mt Ningua, on Boulouparis–Thio Road, about 50 m upstream road, 21°43.613'S, 166°06.567'E, 150 m, 29.xi–12.xii.2003, Malaise trap, loc#054, leg. K.A. Johanson (NHRS); 7 males, Province Sud, stream draining to Rivière des Pirogues, 850 m E summit of Mont Imbaah, 5.5 km E Lucky Creek in Plum, 22°16.837'S, 166°42.195'E, 31 m, 01.xii.2003, light trap, loc#060, leg. K.A. Johanson (NHRS); males, 2 females, Province Nord, Aoupinié Mtn, Réserve spéciale de faune de l’Aoupinié, spring to side stream to Öröpömwati river, 21°09.032'S, 165°19.179'E, 441 m, 6–27.xii.2003, Malaise trap, loc#065, leg. K.A. Johanson (NHRS); 3 males, Province Nord, small stream crossing road RPN3 between Touho and Poindimié, about 200 m S Tiwaka River, 20°49.105'S, 165°15.182'E, 30 m, 6–27.xii.2003, Malaise trap, loc#066, leg. K.A. Johanson (NHRS); numerous males, females, Province Nord, Wemwâdiu stream, 850 m E summit Kögi Mtn, 5 m upstream road, about 200 m S Tiwaka River, 20°49.020'S, 165°14.165'E, 24 m, 6–27.xii.2003, Malaise trap, loc#067, leg. K.A. Johanson (NHRS); numerous males, females, Province Nord, 50 m upstream bridge on Hienghène–Tnèdo road, 3.9 km S summit of Mt Tnèda, 2.2 km E Tnèdo, 20°43.085'S, 164°49.928'E, 29 m, 7.xii.2003, light trap, loc#071, leg. K.A. Johanson (NHRS); 2 males, 3 females, Province Nord, 1 m upstream road, below waterfall on Hienghène–Tnèdo road, 2.2 km SSW summit of Mt Unpac, 4.9 km ESE Tnèdo, 20.73879°S, 164.85508°E, 7.xii.2003, light trap, loc#072, leg. K.A. Johanson (NHRS); numerous males, females, Province Nord, Bouérabate Stream, S Mont Ninndo, along road Barabache–Boulagoma, 20°17.409'S, 164°11.242'E, 60 m, 19.xii.2003–7.i.2004, Malaise trap, loc#089, leg. K.A. Johanson (NHRS); numerous males (4 on slides), females, Province Nord, Rivière Néhoué, camp Amenage de Néhoué, 20°25.037'S, 164° 13.222'E, 12 m, 19.xii.2003, light trap, loc#090, leg. K.A. Johanson (NHRS); numerous males (1 on slide), females, Province Nord, Ponandou Tiôgé River at Kögi, 3.9 km SSW Touho, 20°49.043'S, 165°13.551'E, 25 m, 26.xii.2003, light trap, loc#100, leg. K.A. Johanson (NHRS, ANIC); 5 males, 3 females, Province Nord, Plaine des Gaïacs, Rivière Rouge, 14.2 km NW summit of Mt Rouge, 50 m upstream road RT1 Noumea–Koné, 21°31.573'S, 164°46.690'E, 23 m, 2.i.2004, light trap, loc#104, leg. K.A. Johanson (NHRS); 3 males, Province Sud, Fö Néchédeva stream, 2 m upstream bridge on La Foa–Koindé road, 21°38.812'S, 165°56.076'E, 124 m, 4.i.2004, light trap, loc#106, leg. K.A. Johanson (NHRS); numerous males, females, Province Sud, Sarraméa, Xwê Wya River, 21°38.318'S, 165°51.582'E, 127 m, 17–18.i.2004, light trap, loc#121, leg. K.A. Johanson (NHRS); 4 males Province Sud, stream crossing way to sanatorium 2.3 km E St Laurent, ca. 150 m upstream bridge, 22°04.484'S, 166°19.910'E, loc 027, Malaise trap, 17–19.x.2006, leg. K.A. Johanson M Espeland, (NHRS).

###### Remarks.

*Oxyethira oropedion* is one of the more commonly collected New Caledonian *Oxyethira* species, occurring throughout the island (Fig. 97); several of the collecting sites are shown in photographs of type localities of other species (Figs 75, 76, 79, 81). Kelley’s (1989) illustration of the ventral view of the male exaggerates the separation of the lobes of the gonopods, which was undoubtedly what led Oláh and Johanson (2010a: fig. 50) to interpret the closer position seen in the single specimen on which they based *Oxyethira derek* as indicative of a separate morphospecies. Examination of the type of *Oxyethira oropedion* shows that it shares the features of *Oxyethira derek* and thus we suppress *Oxyethira derek* in synonymy.

##### Oxyethira (Pacificotrichia) quadrata sp. n.

Taxon classificationAnimaliaTrichopteraHydroptilidae

http://zoobank.org/C107DB43-8193-4F74-8635-D96FDB94AFE5

Figs 38 , 98

###### Diagnosis.

This species is distinguished in the male from the similar species *Oxyethira oropedion* by the broadly truncate subgenital process rather than triangular to rounded, flanges on the phallic apparatus; and by the significantly longer antennae of 32 flagellomeres compared to 22–24 flagellomeres.

###### Description.

Male antennae with 32 flagellomeres, flagellomeres rectangular in profile, without *sensilla placodea*; anterior wing length 1.8 mm (n=1); tibial spurs 0,3,4; posterior spurs on hind leg longer than anterior spurs; abdominal sternite VII with sharp spur sub-apico-medially.

Male, genitalia (Fig. 38). Abdominal segment VIII rounded proximally, tapered slightly to distal margin, a pair of small setose lobes mesally at base of gonopods, gonopods in form of short discrete conical lobes, setose lobes of ventral processes short, subgenital process a subquadrate plate. Phallic apparatus distally with a lateral flange, but no free spine or titillator present.

###### Material examined.

**Holotype.** Male (on slide), New Caledonia, Province Sud, Mt Dzumac, source stream of Ouinne River, near crosspoint to mountain track, 22°02.073'S, 166°28.460'E, 810 m, 18.xi–4.xii.2003, Malaise trap, loc#030, leg. K.A. Johanson (MNHP).

###### Etymology.

The name *quadrata* is descriptive of the shape of the subgenital processes.

###### Remarks.

Only one specimen of this species was identified, from the far south of the island (Fig. 98). Were it not for the exceedingly long antennae, we would probably have referred it to *Oxyethira oropedion*, broadening the concept of *Oxyethira oropedion*. A photograph of the type locality with the trap is rendered in Fig. 79.

##### Oxyethira (Pacificotrichia) dorsennus

Taxon classificationAnimaliaTrichopteraHydroptilidae

Kelley

Figs 39 , 40 , 80 , 99

Oxyethira dorsennus Kelley, 1989: 199, figs 35, 44, 58.

###### Revised diagnosis.

Males are distinguished from the closely similar *Oxyethira indorsennus* by having spur formula 0,2,4, apical margins of gonopods truncate and subgenital process triangular rather than rounded as in *Oxyethira indorsennus*. These features also distinguish *Oxyethira dorsennus* from *Oxyethira oropedion*, which has apically well-separated gonopods with a pair of small setose lobes midventrally. Kelley (1989) distinguished *Oxyethira dorsennus* from *Oxyethira indorsennus* on the basis of spur count and the shape of dorsum [abdominal segment] VIII; this latter feature, however, appears to be less reliable than the shape of genital structures.

Male, antennae with 24 flagellomeres, flagellomeres rectangular in profile, without *sensilla placodea*. Anterior wing length, 1.5–2.1 mm (n=2). Tibial spurs 0,2,4. Abdominal sternite VII with short strong spur medially, offset from distal margin.

###### Material examined.

**Holotype.** Male, New Caledonia, mountain stream up Boulari River, (BPBM). **Other material.** 1 male (on slide), Province Sud, Couvelée River at Haute Couvelée, 2.8 km SV summit of Mt Piditéré, 3.5 km NNE Dumbéa, 22°07.405'S, 166°28.023'E, 27 m, 28.xi.2003, light trap, loc#052, leg. K.A. Johanson (NHRS); 1 male (on slide), Province Sud, Xwé Pemöu Stream, 300 m N bridge over Dathio River at Atè, 6.2 km WNW Thio, 21.58835°S, 166.15117°E, 13 m, 29.xi.2003, light trap, loc#056, leg. K.A. Johanson (NHRS); 1 male, Province Sud, lower part Rivière des Pirogues, 800 m WNW summit of Mont Imbaah, 4.7 km E Lucky Creek in Plum, 22°18.559'S 166°41.227'E, 1.3 m, 1.xii.2003, light trap, loc#059, leg. K.A. Johanson (NHRS).

###### Remarks.

Only three further specimens of this species have been identified, all from the south of the island (Fig. 99). They conform in detail with the type. A photograph of the type locality with the trap is rendered in Fig. 80.

##### Oxyethira (Pacificotrichia) indorsennus

Taxon classificationAnimaliaTrichopteraHydroptilidae

Kelley

Figs 41–43 , 100

Oxyethira indorsennus Kelley, 1989: 199, fig. 36.
Oxyethira tompa Oláh & Johanson, 2010a: 98, figs 56–58. **Syn. n.**

###### Revised diagnosis.

In ventral view males of *Oxyethira indorsennus* are distinguished from those of similar species, such as *Oxyethira dorsennus*, *Oxyethira oropedion* and *Oxyethira quadrata* by the rounded apical margins of the gonopods and apically rounded subgenital process, which contrast with the truncate apical margins of gonopods and triangular subgenital process of *Oxyethira dorsennus*; the absence of a pair of small median setose lobes between gonopods and clearly rounded subgenital process distinguish them from *Oxyethira oropedion*; and closely abutting gonopods, rather than widely separated, and rounded subgenital process rather than quadrate separates *Oxyethira indorsennus* from *Oxyethira quadrata*.

Male, antennae with 23–24 flagellomeres, flagellomeres rectangular in profile, without *sensilla placodea*. Anterior wing length, 1.3–2.1 mm (n=10). Tibial spurs 0,3,4. Abdominal sternite VII with sharp median spine, offset from distal margin.

###### Material examined.

*Oxyethira indorsennus* Kelley, **Holotype.** male, New Caledonia, mountain stream up Boulari River, (BPBM).

###### Other material.

1 Male, Province Sud., Rivière Bleue, 282 m, stony river, loc 4, 22°05.705'S, 166°38.225'E, Malaise trap, 13–16.xi.2001, leg. K.A. Johanson, T. Pape & B. Viklund(NHRS); 1 male (on slide), 6 females (1 on slide), Province Sud, stony stream draining Lac Yaté, 200 m, loc 5, 22°08.795'S, 166°42.313'E, Malaise trap 13–16.xi.2001, leg. K.A. Johanson, T. Pape & B. Viklund(NHRS); 11 males, 5 females, Province Nord, Mt Acupinié, fauna reserve, 482 m, stream, loc 19, 2109.369'S, 16519.209'E, Malaise trap, 24–28.xi.2001, leg. K.A. Johanson, T. Pape & B. Viklund(NHRS); 10 males, 12 females, Province Sud, stream draining to Marais de la Rivière Blanche, 1.35 km S Pont Pérignon, 22°08.496'S, 166°42.152'E, 180 m, 6–16.xi.2003, Malaise trap, loc#009, leg. K.A. Johanson (NHRS); numerous males, females, Province Sud, stream draining to Marais de la Rivière Blanche, 2.25 km SW Pont Pérignon, 22.14158°’S, 166.67993 °E, 157 m, 6–16.xi.2003, Malaise trap, loc#010, leg. K.A. Johanson (NHRS); 14 males, Province Sud, side stream to Rivière Blanche, 10.75 km SW Pont Pérignon, 22°10.073'S, 166°39.903'E, 180 m, 6–16.xi.2003, Malaise trap, loc#012, leg. K.A. Johanson (NHRS); 2 males, Province Sud, Monts Kwa Ne Mwa, on road between Noumea and Yaté, 2.0 km E Pic Mouirange, 22°12.356'S, 166°40.798'E, 220 m, 7–16.xi.2003, Malaise trap, loc#014, leg. K.A. Johanson (NHRS); numerous males, Province Sud, W slope Mt Ningua, Kwé Néco Stream, 3.9 km W summit of Mt Ningua, on Boulouparis–Thio Road, about 50 m upstream road, 21°44.359'S, 166°06.009'E, 117 m, 20.xi–12.xii.2003, Malaise trap, loc#035, leg. K.A. Johanson (NHRS); 1 male, Province Sud, Platou de Dogny, source of Dogny River, about 900 m SE summit of Platou de Dogny, 21.61917°S, 165.88072°E, 919 m, 25.xi–16.xii.2003, Malaise trap, loc#046, leg. K.A. Johanson (NHRS); 1 male, Province Sud, W slope Mt Ningua, Kwé Néco Stream, at Camp Jacob, 3.9 km W summit of Mt Ningua, on Boulouparis–Thio Road, about 50 m upstream road, 21°44.083'S, 166°06.298'E, 117 m, 29.xi.2003–12.xii.2003, Malaise trap, loc#053, leg. K.A. Johanson (NHRS); 8 males (2 on slides), Province Sud, W slope Mt Ningua, Kwé Néco Stream, at Camp Jacob, 3.7 km WNW summit of Mt Ningua, on Boulouparis–Thio Road, about 50 m upstream road, 21°43.613'S, 166°06.567'E, 150 m, 12.xii.2003–05.i.2004, Malaise trap, loc#054, leg. K.A. Johanson (NHRS); 54 males, 18 females, Province Nord, Mt Panié, stream at camp, 20.58167°S, 164.76472°E, 1311 m, 9.xii.2003, Malaise trap, loc#073, leg. K.A. Johanson (NHRS); numerous males, females, Province Nord, Mt Panié, stream at camp, 20. 58139°S, 164.76444°E, 1310 m, 9.xii.2003–2.i.2004, Malaise trap, loc#074, leg. K.A. Johanson (NHRS); 4 males, Province Nord, stream in Creek de Bambou, 5 m N road RT7 Ouégoa–Koumac, 20°27.863'S, 164°19.784'E, 58 m, 19.xii.2003, Malaise trap, loc#087, leg. K.A. Johanson (NHRS); 36, 15 females, Province Nord, Bouérabate Stream, S Mont Ninndo, along road Barabache–Boulagoma, 20°17.409'S, 164°11.242'E, 60 m, 19.xii.2003–7.i.2004, Malaise trap, loc#089, leg. K.A. Johanson (NHRS); 2 males, Province Sud, Co Rigule Stream, 2.1 km N bridge over Baie de Yaté, 4.3 km S Wé Ngéré, 22°08.147'S, 166° 56.072'E, 14 m, 18.i.2004, light trap, loc#122, leg. K.A. Johanson (NHRS).

###### Remarks.

Kelley (1989: 199, fig. 36) distinguished *Oxyethira indorsennus* from *Oxyethira dorsennus* on the basis of difference in spur formula (0,3,4 cf. 0,2,4) and “… shape of dorsum VIII”. However, examination of the types shows *Oxyethira indorsennus* to differ also in shape of the apical margins of gonopods and subgenital process, these being rounded as illustrated for *Oxyethira tompa* which also shares features such as spur formula and number of antennal flagellomeres (=24). Thus we are synonymising *Oxyethira tompa* with *Oxyethira indorsennus*. The species was collected widely throughout the island (Fig. 100).

This species shows a general resemblance to *Oxyethira smolpela* Wells, from New Guinea, but that species has a distinctive titillator on the phallic apparatus which is lacking in *Oxyethira indorsennus*.

##### Oxyethira (Pacificotrichia) rougensis sp. n.

Taxon classificationAnimaliaTrichopteraHydroptilidae

http://zoobank.org/9B1D6AF1-0FEC-41A1-8DD1-E5F4A3591C32

**Figures 44–52.**
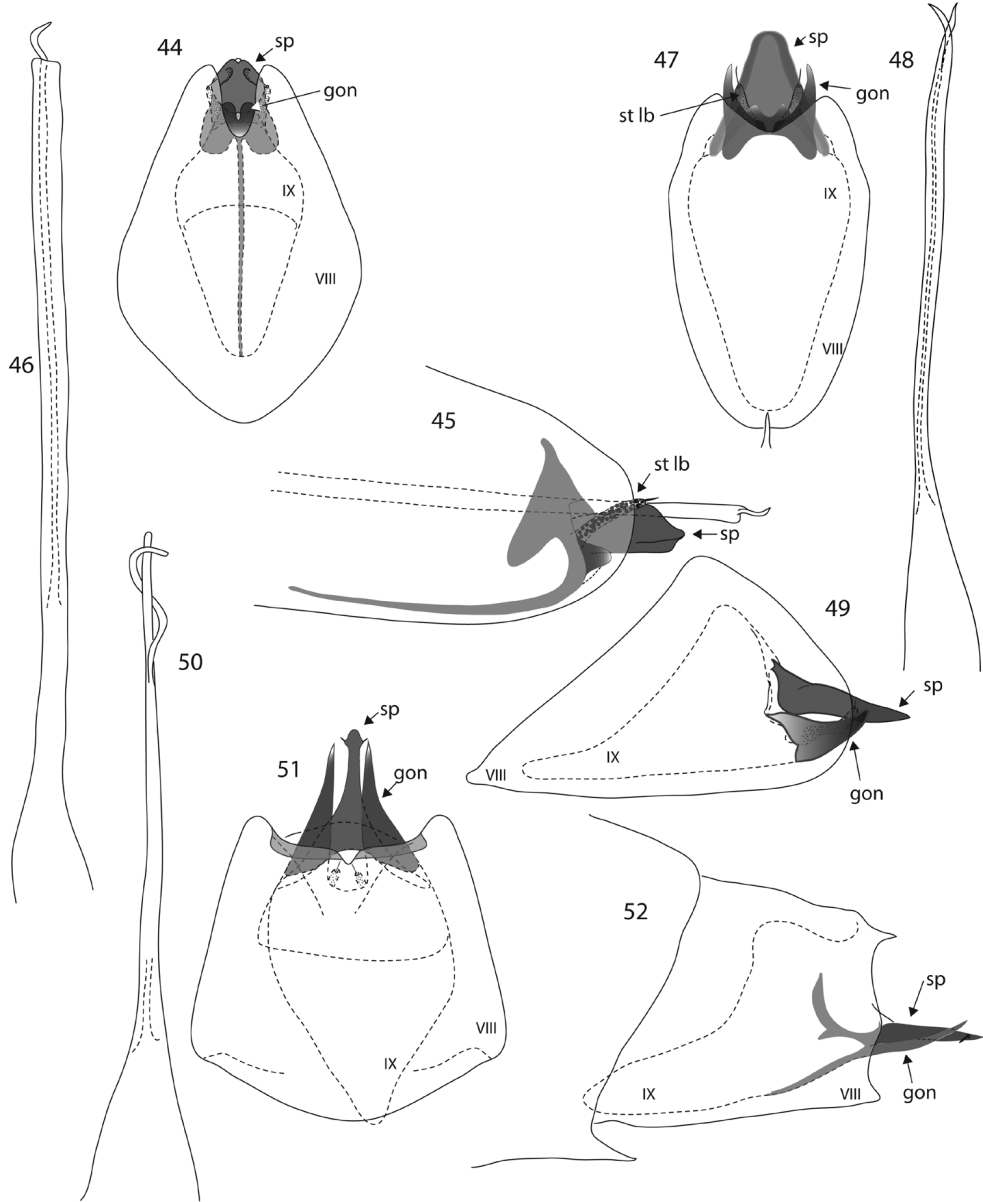
*Oxyethira* species male genitalia. **44–46** *Oxyethira rougensis* sp. n., ventral and lateral views and phallic apparatus **47–49** *Oxyethira mouirange* sp. n., ventral view, phallic apparatus and lateral view **50–52** *Oxyethira ouenghica* sp. n., phallic apparatus, ventral and lateral views. Abbreviations: gon = gonopod; sp = subgenital process; st lb = setose lobe of subgenital process; VIII, IX = abdominal segments VIII and IX.

Figs 44–46 , 81 , 101

###### Diagnosis.

*Oxyethira rougensis* sp. n. groups with *Oxyethira oropedion*, *Oxyethira quadrata*, *Oxyethira dorsennus*, and *Oxyethira indorsennus*, but unlike any of those species, males have venter of abdominal segment VIII deeply and narrowly excised apico-medially and subgenital process sculpted latero-distally.

###### Description.

Male antennae: with 23–24 flagellomeres, flagellomeres rectangular in profile; anterior wing length 1.1–1.8 mm (n=3); tibial spurs 0,3,4; abdominal sternite VII with short sharp median spine on distal margin.

Male, genitalia. Abdominal segment VIII conical, distally about half width of proximal quarter. Segment IX in ventral view almond shaped, in lateral view triangular proximally, mid-dorsally less than half length of venter. Gonopods fused basally, discrete and rounded distally, with slender mid-ventral apodeme reaching to proximal margin of segment IX; subgenital processes fused, forming a stout plate, rounded distally in ventral view, angular in lateral view, with subapical sculpturing towards apex on each side, and small notch mid apically; setose lobes about 2/3 length of plate. Phallic apparatus slender, straight, with short apical spine.

###### Material examined.

**Holotype.** Male (on slide), New Caledonia, Province Nord, Plaine des Gaïacs, Rivière Rouge, 14.2 km NW summit of Mt Rouge, 50 m upstream road RT1 Noumea–Koné, 21°31.573'S, 164°46.690'E, 23 m, 2.i.2004, light trap, loc#104, leg. K.A. Johanson (NHRS).

**Paratypes.** 2 males (on slides), collection data as for holotype.

###### Remarks.

*Oxyethira rougensis* was taken only at the type locality, in the north-west of the island (Fig. 101). A photograph of the type locality with the trap immediately below the small waterfall is rendered in Fig. 81.

##### Oxyethira (Pacificotrichia) mouirange sp. n.

Taxon classificationAnimaliaTrichopteraHydroptilidae

http://zoobank.org/323D9AB1-425C-4A4D-9B82-3DAD6EF9CBE0

Figs 47–49 , 102

###### Diagnosis.

A member of *Pacificotrichia* subgenus, but distinguished from other species by males with discrete, elongate, sub-triangular gonopods.

###### Description

**male.** Antennae with 24–25 flagellomeres; flagellomeres without *sensilla placodea*, rectangular in profile; anterior wing length 1.6–1.7 mm (n=2); tibial spurs 0,3,4; abdominal sternite VII with small sharp median spur.

Male, genitalia. In ventral view with abdominal segment VIII ovoid, apical margin with wide V-shaped excavation medially; without midventral apodeme; in lateral view, with apical margins broadly rounded; abdominal segment IX almond shaped. Gonopods sharply triangular; subgenital processes fused, forming subtriangular plate that is sharply triangular in lateral view; setose lobes less than half length of plate. Phallic apparatus extremely long, apically bifid, split into two equal-length sections.

###### Material examined.

**Holotype.** Male (on slide), New Caledonia, Province Sud, Monts Kwa Ne Mwa, on road between Noumea and Yaté, 2.0 km E Pic Mouirange, 22°12.356'S, 166°40.798'E, 220 m, 7–16.xi.2003, Malaise trap, loc#014, leg. K.A. Johanson (MNHP).

**Paratypes.** 2 males (on slides, one headless), collection data as for holotype (NHRS); 1 male, Province Sud, Sarraméa, Xwê Wya River, 21°38.318'S, 165°51.582'E, 127 m, 17–18.i.2004, light trap, loc#121, leg. K.A. Johanson (NHRS).

###### Etymology.

Named for Mt Mouirange near which the type was collected.

###### Remarks.

Taken only at two well-separated sites in the south of the island (Fig. 102).

##### Oxyethira (Pacificotrichia) ouenghi sp. n.

Taxon classificationAnimaliaTrichopteraHydroptilidae

http://zoobank.org/79A15B02-C3D1-4175-A9A4-539A06DE8894

Figs 50–52 , 75 , 103

###### Diagnosis.

Males resemble *Oxyethira nehoue* sp. n., *Oxyethira melasma*, and *Oxyethira spicula* sp. n. in having more or less triangular median ventral processes in the male genitalia. However, males of *Oxyethira ouenghi* differ from other New Caledonian species, having gonopods in form of slender curved spines adjacent to the narrow midventral process.

###### Description

**male.** Antennae with 23–25 flagellomeres, each flagellomere length greater than width, without *sensilla placodea*; Anterior wing length 1.7–1.8 mm (n=2); tibial spurs 0,3,4; abdominal sternite VII with mid apical spine.

Male, genitalia (Figs 50–52). Abdominal segment VIII tapered slightly towards apex, only slightly longer than wide, with small cleft apico-ventrally and short apico-lateral lobes; in ventral view abdominal segment IX broadest mid length, tapered sharply proximally and distally, with gonopods forming a pair of narrow curved spines closely adpressed to an elongate triangular ventral process that terminates with a pair of tiny setae; phallic apparatus elongate, slender, with a long fine sinuous subapical process.

###### Material examined.

**Holotype.** Male (on slide), New Caledonia, Province Nord, Bouérabate Stream, S Mont Ninndo, along road Barabache–Boulagoma, 20°17.409'S, 164°11.242'E, 60 m, 19.xii.2003–7.i.2004, Malaise trap, loc#089, leg. K.A. Johanson (MNHP).

**Paratypes.** 8 males, same data as for holotype; 1 male (on slide), Ouenghi River, nr Boulouparis, 14.xii.1983, A Wells (ANIC); 1 male, Province Sud, Monts Kwa Ne Mwa, on road between Noumea and Yaté, 2.0 km E Pic Mouirange, 22°12.356'S, 166°40.798'E, 220 m, 7–16.xi.2003, Malaise trap, loc#014, leg. K.A. Johanson (NHRS); 1 male (on slide), Province Sud, Monts des Koghis, ca 800 m S Koghi Restaurant, 22.18311°S, 166.50564°E, 460 m, 10–26.xi.2003, Malaise trap, loc#019, leg. K.A. Johanson (NHRS); 3 males, Province Sud, Mt Dzumac, source stream of Ouinne River, near crosspoint to mountain track, 22°02.073'S, 166°28.460'E, 810 m, 18.xi–4.xii.2003, Malaise trap, loc#030, leg. K.A. Johanson (NHRS).

###### Etymology.

Named for the Ouenghi River beside which the first specimen was collected.

###### Remarks.

The apparent distribution of this very distinctive species is very odd, with one sample being taken in the extreme north of the island, the rest at sites in the south-west (Fig. 103). A photograph of the type locality with the trap is rendered in Fig. 75.

##### Oxyethira (Pacificotrichia) enigmatica sp. n.

Taxon classificationAnimaliaTrichopteraHydroptilidae

http://zoobank.org/BFA21817-6E77-4056-BD89-45C2483C51F3

**Figures 53–60.**
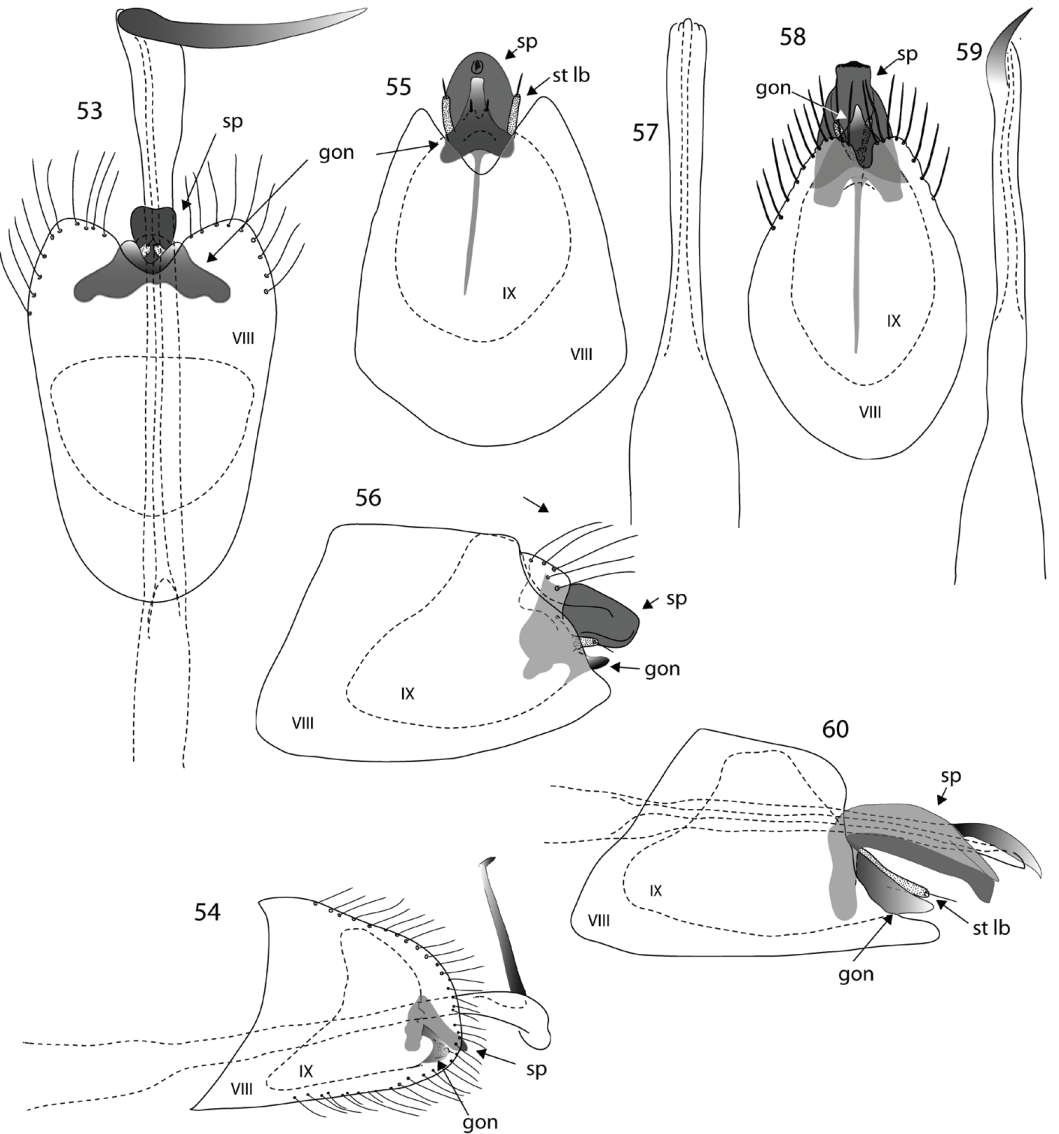
*Oxyethira* species male genitalia. **53, 54** *Oxyethira enigmatica* sp. n., ventral and lateral views **55–57** *Oxyethira melasma* Kelley, ventral and lateral views, and phallic apparatus, drawn from holotype **58–60** *Oxyethira nehoue* sp. n., ventral view, phallic apparatus and lateral view. Abbreviations: gon = gonopod; sp = subgenital process; st lb = setose lobe of subgenital process; VIII, IX = abdominal segments VIII and IX.

Figs 53–54 , 82 , 104

###### Diagnosis.

In having an elongate apical spine on the phallic apparatus, males of this species resemble those of *Oxyethira scutica*, but in *Oxyethira enigmatica* sp. n. the spine is shorter and strap-like, not thread-like as in *Oxyethira scutica*; abdominal segment VIII tapers and gradually increases in width distally, and distal margin of venter is more widely and shallowly excavated than in *Oxyethira scutica* in which it is deeply and narrowly excised.

###### Description.

Male antennae with 23–24 flagellomeres, flagellomeres without *sensilla placodea*, in profile almost subquadrate, but wider apically than proximally; anterior wing length, 1.5–1.6 mm (n=10); tibial spurs 0,2,4; abdominal segment VII with a sharp spur medially on distal margin.

Male, genitalia (Figs 53–54). Abdominal segment IX in ventral view wider distally than proximally, with distal margin shallowly excavated, proximal margin rounded, midventral apodeme present; in lateral view dorsal margin about half depth of ventral margin. Gonopods fused basally, distally discrete, conical; subgenital processes forming short, subquadrate plate. Phallic apparatus straight, with long sharply twisted apical spine, at right angle to and almost one third length of phallus; without titillator.

###### Material examined.

**Holotype.** Male (on slide), Province Sud, W part of Plaine des lacs, 150 m downstream bridge at La Capture, 22°15.967'S, 166°49.493'E, 261 m, 4–22.xi.2003, Malaise trap, loc#007, leg. K.A. Johanson (MNHP).

**Paratypes.** 54 males (9 on slides), data as for holotype.

###### Etymology.

*Enigmatica*: enigmatic in presenting a puzzle, being so similar to Oxyethira (Pacificotrichia) scutica in some respects, yet distinctive.

###### Remarks.

This species is known only from the large sample taken at the type locality in the extreme south of the island (Fig. 104). A photograph of the type locality with the trap is rendered in Fig. 82.

##### Oxyethira (Pacificotrichia) melasma

Taxon classificationAnimaliaTrichopteraHydroptilidae

Kelley

Figs 55–57 , 105

Oxyethira melasma Kelley, 1989: 200, figs 37, 45, 59.

###### Note.

A mismatch between the published description and Kelley’s (1989) figures and the holotype specimen in the BPBM labelled “*Oxyethira melasma*” is a puzzle. The genital features of the holotype (examined by AW) are as in Fig. 56, and disagree with Kelley’s text description and illustrations (his figs 37, 45, 59) of “*Oxyethira melasma*”. However, the tibial spur count of the holotype is 0,2,4, as given by Kelley, and as in the type species. Kelley’s three figures of genital structures agree with the features of a series of specimens, described here as *Oxyethira nehoue* and illustrated in Figs 58–60; in contrast to Kelley’s “holotype”, these specimens all have a tibial spur count of 0,3,4.

*Oxyethira melasma* Kelley is here redescribed and figured from the holotype specimen. We can only suggest that Kelley had several specimens at hand, macerated one and labelled it “holotype”, but described and illustrated a specimen other than that labelled “Type”. The type has to be the name-bearing specimen and is redescribed here.

###### Revised diagnosis.

Males resemble *Oxyethira nehoue* sp. n., *Oxyethira spicula* sp. n., and *Oxyethira ouenghi* in having have a more or less triangular median ventral processes in the male genitalia. However, they most closely resemble *Oxyethira nehoue* sp. n. from which they are distinguished by spur count 0,2,4, gonopods fused, in ventral view sharply tapered proximally, narrowly parallel-sided distally, rounded apically, apex of phallic apparatus rounded without apical spine, in contrast to *Oxyethira nehoue* sp. n. in which the fused gonopods appear triangular in ventral view, and acuminate apically, and ventral process in lateral view sclerotised and arched ventrally.

###### Revised description

**male.** Antennae with 19–26 flagellomeres, apical 3 flagellomeres pale, next 5 dark, then 13 pale and basal flagellomeres dark; anterior wing length 1.4–1.9 mm (n=10); tibial spurs 0,2,4; abdominal sternite VII with small sharp median spine, offset from margin.

Male, genitalia (Figs 55–57). Abdominal segment VIII more or less conical, a deep broadly V-shaped excision apico-ventrally. Abdominal segment IX in lateral view broadly bell-shaped; in ventral view rounded proximally, tapered distally, a pair of small short processes apically, each bearing a single seta. Gonopods sclerotised, fused, tapered to narrowly truncate apex, basal setose processes widely separated, slender, elongate, but shorter than fused gonopods, a slender basal apodeme midventrally; subgenital plate broad based, tapered to rounded apex, mostly membranous, but with a small ventrally curved prominence subapically. Phallic apparatus swollen in basal third, narrow in distal 2/3, without apical spine, ejaculatory tube medial.

###### Material examined.

**Holotype.** Male, Mountain stream up Boulari River (BPBM). **Other material.** 18 males, Province Sud, W part of Plaine des lacs, 150 m downstream bridge at La Capture, 22°15.967'S, 166°49.493'E, 261 m, 04–22.xi.2003, Malaise trap, loc#007, leg. K.A. Johanson (NHRS); 31males, Province Sud, Mt Dzumac, source stream of Ouinne River, near crosspoint to mountain track, 22°02.073'S, 166°28.460'E, 810 m, 18.xi–4.xii.2003, Malaise trap, loc#030, leg. K.A. Johanson (NHRS); 1 male, Province Sud, Mt Dzumac, source stream of Ouinne River, downstream crosspoint to mountain track, 22°01.997'S, 166°28.486'E, 795 m, over about 30 m waterfall, 18.xi–4.xii.2003, Malaise trap, loc#031, leg. K.A. Johanson (NHRS); 1 male (on slide), Province Sud, W slope Mt Ningua, Kwé Néco, Stream, at Camp Jacob, 3.7 km WNW summit of Mt Ningua, on Boulouparis–Thio Road, about 50 m upstream road, 21°43.613'S, 166°06.567'E, 150 m, 29.xi–12.xii.2003, Malaise trap, loc#054, leg. K.A. Johanson (NHRS); 4 males, Province Sud, Co Rigule Stream, 2.1 km N bridge over Baie de Yaté, 4.3 km S Wé Ngéré, 22°08.147'S, 166° 56.072'E, 14 m, 18.i.2004, light trap, loc#122, leg. K.A. Johanson (NHRS).

###### Remarks.

The species was taken at a number of sites in the southern province of the island (Fig. 105).

##### Oxyethira (Pacificotrichia) nehoue sp. n.

Taxon classificationAnimaliaTrichopteraHydroptilidae

http://zoobank.org/EE2F9749-F988-4653-9556-D40FFD285635

Figs 58–60 , 83 , 106

###### Diagnosis.

This species resembles *Oxyethira melasma* and was illustrated and described as that species by Kelley (1989); however the holotype is as in Figs 55–57, see discussion above. The two species are distinguished by male genital features: *Oxyethira nehoue* sp. n. has abdominal segment VIII in ventral view shallowly and narrowly excavated mid apically, not widely and deeply; fused gonopods tapered to an acute apex, not truncate; subgenital process elongate rectangular, truncate apically, not rounded, in lateral view sclerotised and arched ventrally; and phallic apparatus with a broad spine arising sub apically.

###### Description.

Male antennae with 25–27 flagellomeres; anterior wing length 1.3–1.7 mm (n=7); tibial spurs 0,3,4; abdominal sternite VII with sharp apical spine.

Genitalia (Figs 58–60). Abdominal segment VIII in ventral view rounded proximally, gradually tapered towards apex, with a shallow, narrow excavation mid apically. Abdominal segment IX similar in shape to VIII. Gonopods fused, triangular in ventral view, dorsal setose lobes slightly shorter than gonopods conjoined at base, subgenital process elongate, forming narrowly rectangular plate, apically truncate, but with slight bulge mid-apically. Phallic apparatus with a broad spine arising subapically, extending beyond apex.

Female unknown.

###### Material examined.

**Holotype.** Male (on slide), Province Nord, Rivière Néhoué, camp Amenage de Néhoué, 20°25.037'S, 164°13.222'E, 12 m, 19.xii.2003, light trap, loc#090, leg. K.A. Johanson (MNHP).

**Paratypes.** 5 males (on slides), data as for holotype (NHRS); 1 male, Province Sud, creek on road between Sarraméa & La Foa, 15.xii.1983, A. Wells (ANIC); 1 male (on slide), Province Sud, stream NE turnoff to Tribu Kouraga on Boulouparis-Thio road, 19.xii.1983, AW (ANIC); 1 male, Ouenghi River, nr Boulouparis, 20.xii.1983, A. Wells (ANIC); 1 male (on slide), Province Sud, NW Farino on Moindou road, 20.vii.1998, AW (ANIC); 20 males, Province Sud, Tamoa River, 700m S road RT1 between Noumea and La Foa, 22°04.518'S, 166°16.592'E, 19.xi.2003, light trap, loc#033, leg. K.A. Johanson (NHRS).

###### Etymology.

Named for the Rivière Néhoué where it was collected.

###### Remarks.

The distribution of this species is similar to that of *Oxyethira ouenghi*, mainly collected from a cluster of southern sites, but with one site in the far north (Fig. 106). A photograph of the type locality with the trap is rendered in Fig. 83.

##### Oxyethira (Pacificotrichia) scutica

Taxon classificationAnimaliaTrichopteraHydroptilidae

Kelley

Figs 61–63 , 107

Oxyethira scutica Kelley, 1989: 200, figs 39, 47, 52, 53, 61.

###### Diagnosis.

Males superficially resemble those of *Oxyethira enigmatica*, having a long apical process on the phallic apparatus, antennae with 23–25 flagellomeres, and spur formula of 0,2,4, but are distinguished by apical process on phallic apparatus longer and thread-or whip-like in appearance, rather than strap-like as in *Oxyethira enigmatica*, and abdominal segment VIII with distal margin of venter of deeply and narrowly excised, compared with shallowly excavated margin of *Oxyethira enigmatica*. Females resemble those of *Oxyethira oropedion* (Kelley 1989: figs 50, 51, both having abdominal terminalia in form of a slender oviscapt and cerci slender, elongate, about length of segment X, but females of *Oxyethira scutica* lack the darkly sclerotised area on the venter of segment IX (Kelley 1989: figs 52, 53).

Antennae: male with 21–24 flagellomeres, flagellomeres subquadrate in profile, without sensilla placodea; female with 18 flagellomeres. Fore wing length: male 1.1–1.5 mm (n=8), female 1.4 mm (n=2). Spurs 0,2,4. Abdominal sternite VII with sharp median spur.

**Figures 61–66.**
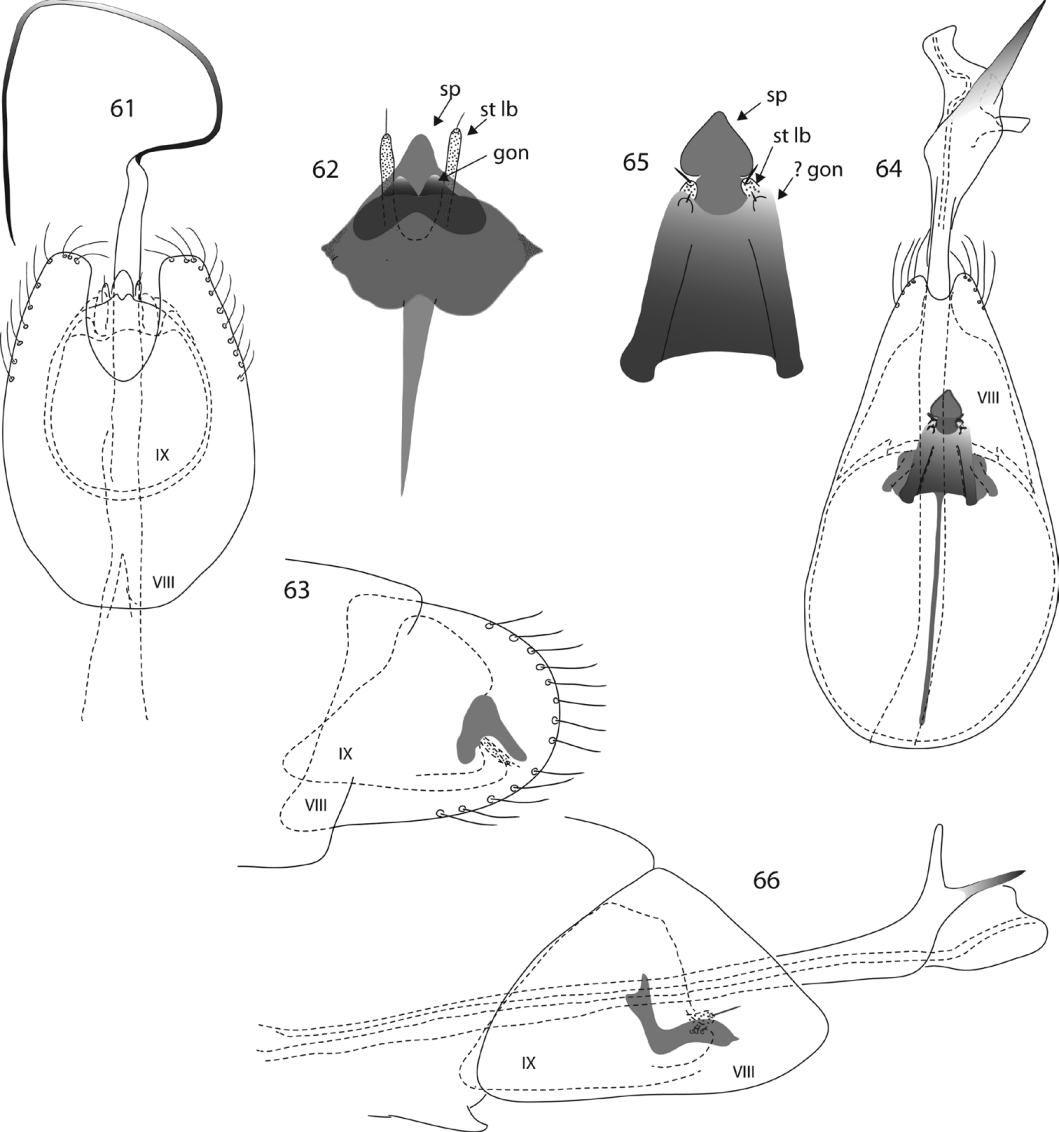
*Oxyethira* species male genitalia. **61–63** *Oxyethira scutica* Kelley, ventral view and enlarged gonopods and subgenital process, and lateral view **64–66** *Oxyethira spicula* sp. n., ventral view and enlarged gonopods and subgenital process, and lateral view. Abbreviations: gon = gonopod; sp = subgenital process; st lb = setose lobe of subgenital process; VIII, IX = abdominal segments VIII and IX.

###### Material examined.

**Holotype.** Male, New Caledonia, mountain stream up Boulari River, (BPBM). **Other material.** 1 male (on slide), Province Sud, Ouenghi River, Boulouparis, 19.xii,1983, A Wells, (ANIC); 1 male (on slide), Province Sud, side stream to Rivière Blanche, 10.75 km SW Pont Pérignon, 22°10.073'S, 166°39.903'E, 180 m, 6–16.xi.2003, Malaise trap, loc#012, leg. K.A. Johanson (NHRS); 7 males, Province Sud, Monts Kwa Ne Mwa, on road between Noumea and Yaté, 1.5 km E Pic Mouirange, 22°12.545'S, 166°40.246'E, 143 m, 9.xi.2003, light trap, loc#018, leg. K.A. Johanson (NHRS); 2 males, Province Sud, Mt Dzumac, source stream of Ouinne River, at crosspoint to mountain track, 22°02.218'S, 166°28.566'E, 797 m, 18.xi.2003, light trap, loc#032, leg. K.A. Johanson (NHRS); 1 male, 1 female, Province Sud, on road between Noumea and Yaté, 1.0 km NW Pont des Japonais, 22°11.427'S, 166°42.868'E, 113 m, 22.xi–4.xii.2003, Malaise trap, loc#039, leg. K.A. Johanson (NHRS); 2 male, 1 female, Province Sud, lower part Rivière des Pirogues, 800 m WNW summit of Mont Imbaah, 4.7 km E Lucky Creek in Plum, 22°18.559'S, 166°41.227'E, 1.3 m, 01.xii.2003, light trap, loc#059, leg. K.A. Johanson (NHRS); 10 males, Province Sud, stream draining to Rivière des Pirogues, 850 m E summit of Mont Imbaah, 5.5 km E Lucky Creek in Plum, 22°16.837'S, 166°42.195'E, 31 m, 01.xii.2003, light trap, loc#060, leg. K.A. Johanson (NHRS).

###### Remarks.

This appears to be another southern species (Fig. 107).

##### Oxyethira (Pacificotrichia) spicula sp. n.

Taxon classificationAnimaliaTrichopteraHydroptilidae

http://zoobank.org/D92127AE-797C-47BD-BDF8-28B57C2D09A7

Figs 64–66 , 108

###### Diagnosis.

Males are most similar to *Oxyethira melasma*, *Oxyethira nehoue* and *Oxyethira ouenghi* all of which have more or less triangular median ventral processes in the male genitalia, but can be recognised by the expanded apex of the phallic apparatus with a prominent acute spine, the very long, proximally rounded, abdominal segment VIII that tapers distally and completely obscures segment IX, and the shape of the plate formed from fused gonopods and subgenital processes.

###### Description.

Male antennae damaged, at least with 19 flagellomeres, flagellomeres rectangular in profile, without *sensilla placodea*; anterior wing length 1.9 mm (n=1); tibial spurs 0,3,4; abdominal sternite VII with a coarse spur medially.

Male, genitalia (Figs 64–66). Abdominal segment VIII pear-shaped, ventrally with narrow distal margin excavated apically; abdominal segment IX obscured by VIII; gonopods fused forming a triangular plate ventrally with subgenital process a short knob-shaped lobe apically and a deep mid ventral apodeme reaching almost full length of segment; phallic apparatus elongate and slender in proximal 3/4, irregularly dilated distally, with sharp apical spine and short lateral process.

Female unknown, although a single unknown female of an *Oxyethira* species was collected with the holotype and could be of this species. The terminalia of this female are slender and elongate, of the form seen in *Oxyethira oropedion* and *Oxyethira scutica*, with a V-shaped marking ventrally on abdominal segment VIII.

###### Material examined.

**Holotype.** Male (slide), New Caledonia, Province Sud, Rivière des Lacs, 1.1 km NW Lac en Huit, 4.9 km NW summit of Pic du Grand Kaori, 22°15.195'S, 166°52.178'E, 10.xii.2003, light trap, loc#078, leg. K.A. Johanson (MNHP).

###### Etymology.

Name being descriptive of the spicule-like spine on the phallic apparatus.

###### Remarks.

This species is known only from the type specimen, collected in the far south (Fig. 108) and now on a prepared slide. It is impossible to know if the state of abdominal segment IX of the type is the usual situation, or simply unusual that segment has retracted in this particular specimen. Regardless, the form of the phallic apparatus is highly distinctive.

##### Oxyethira (Pacificotrichia) digitata sp. n.

Taxon classificationAnimaliaTrichopteraHydroptilidae

http://zoobank.org/7FE76CC0-F62E-471F-B616-2B9BC4724A78

**Figures 67–74.**
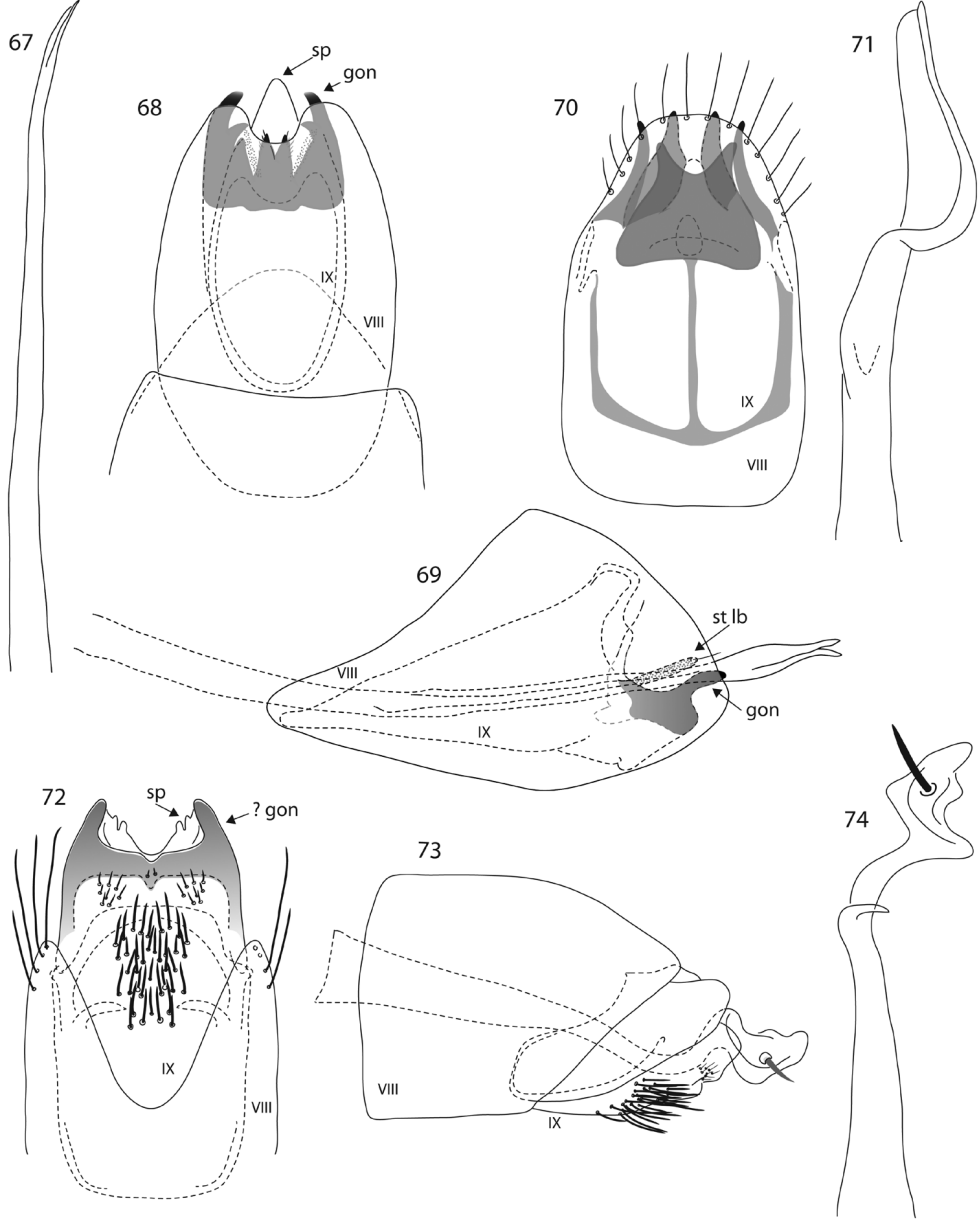
*Oxyethira* species male genitalia. **67–69** *Oxyethira digitata* sp. n., phallic apparatus, ventral and lateral views **70, 71** *Oxyethira incana* (Ulmer), ventral view and phallic apparatus **72–74** *Oxyethira macropennis* sp. n., ventral and lateral views and phallic apparatus. Abbreviations: gon = gonopod; sp = subgenital process; st lb = setose lobe of subgenital process; VIII, IX = abdominal segments VIII and IX.

Figs 67–69 , 84 , 109

###### Diagnosis.

One of the New Caledonian species of *Oxyethira* with genitalia retracted within the very rounded abdominal segment VIII but differs from other species with this feature such as *Oxyethira incana* and *Oxyethira spicula* by well-defined clearly branched gonopods and the phallic apparatus a simple rod, sharply bifid apically.

###### Description.

Male antennae with 25–29 flagellomeres; flagellomeres slender rectangular in profile, without *sensilla placodea*, terminal 5 flagellomeres pale, followed by 3 dark, 10 pale, rest dark; anterior wing. Length 3.0–3.7 mm (n=4); tibial spurs 0,3,4; abdominal segment VII short, sharp mid ventral spur.

Male, genitalia (Figs 67–69). Abdominal segment VIII broadly rounded, ventrally and dorsally concave apically; abdominal segment IX in ventral view rounded, sharply triangular in lateral view, retracted within VIII; gonopods fused basally, distally stoutly bilobed, subgenital process V-shaped, fused ventrally with gonopods; phallic apparatus a simple elongate rod, distally forming sharply bifid apex.

###### Material examined.

**Holotype.** Male (on slide), Province Sud, side stream to Rivière Blanche, 10.75 km SW Pont Pérignon, 22°10.073'S, 166°39.903'E, 180 m, 6–16.xi.2003, Malaise trap, loc#012, leg. K.A. Johanson (MNHP).

**Paratypes.** 6 males (on slides), same data as holotype (NHRS); 1 male Province Sud, stream draining to Marais de la Rivière Blanche, 1.35 km S Pont Pérignon, 22°08.496'S, 166°42.152'E, 180 m, 6–16.xi.2003, Malaise trap, loc#009, leg. K.A. Johanson (NHRS); 1 male, Province Sud, Monts Kwa Ne Mwa, on road between Noumea and Yaté, Rivière des Pirogues, 22°11.225'S, 166°43.338'E, 100 m, 7.xi.2003, light trap, loc#016, leg. K.A. Johanson (NHRS); 1 male (on slide), Province Sud, Mt Dzumac, source stream of Ouinne River, near crosspoint to mountain track, 22°02.073'S, 166°28.460'E, 810 m, 18.xi–4.xii.2003, Malaise trap, loc#030, leg. K.A. Johanson (NHRS); 1 male (on slide), Province Sud, W slope Mt Ningua, Kwé Néco, Stream, at Camp Jacob, 3.7 km WNW summit of Mt Ningua, on Boulouparis–Thio Road, about 50 m upstream road, 21°43.613'S, 166°06.567'E, 150 m, 29.xi–12.xii.2003, Malaise trap, loc#054, leg. K.A. Johanson (NHRS).

###### Etymology.

Named for the finger-like lobes of the gonopods in male.

###### Remarks.

*Oxyethira digitata* shares a southern distribution (Fig. 109) with *Oxyethira melasma* and *Oxyethira scutica*. A photograph of the type locality with the trap is rendered in Fig. 84.

**Figures 75–80.**
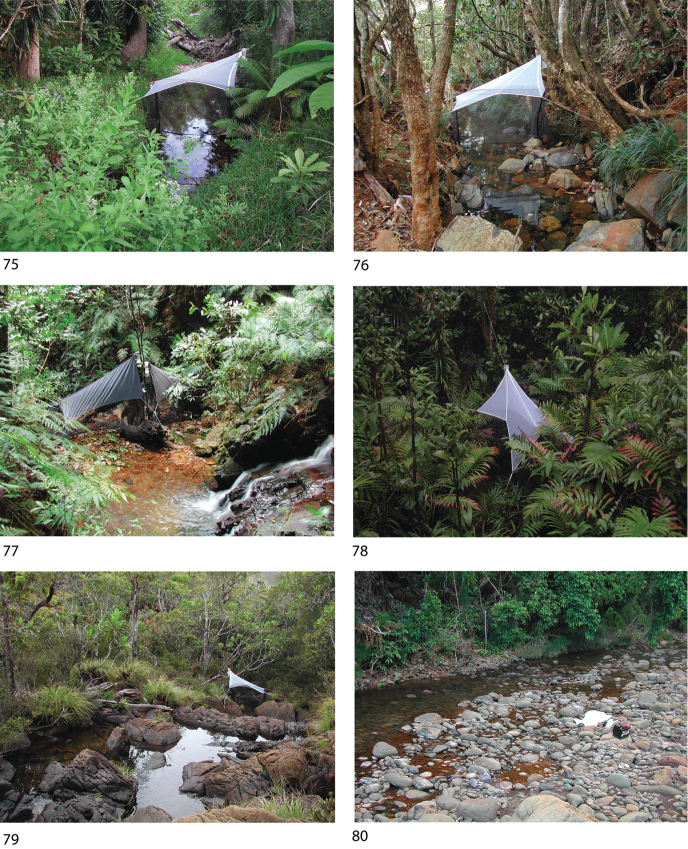
Type localities of *Oxyethira* species. **75** *Oxyethira tiwaka* sp. n. and *Oxyethira ouenghi* sp. n. (collected together with the hydroptilid species *Oxyethira caledonensis*, *Oxyethira oropedion*, *Oxyethira indorsennus*, *Oxyethira incana*, *Hydroptila losida*, *Hellyethira malleoforma*, *Acritoptila disjuncta* Kelley, 1989, *Acritoptila crinita* Kelley, 1989, *Acritoptila glossocercus* Kelley, 1989 and *Acritoptila amphapsis* Kelley, 1989) **76** *Oxyethira perignonica* sp. n. (collected together with the hydroptilid species *Oxyethira oropedion*) **77** *Oxyethira abbreviata* sp. n. (no other Hydroptilidae species collected at this site) **78** *Oxyethira incurvata* (collected together with the hydroptilid species *Oxyethira caledonensis*) **79** *Oxyethira parinsularis* sp. n. and *Oxyethira quadrata* sp. n. (collected together with the hydroptilid species *Oxyethira incurvata* sp. n., *Oxyethira caledonensis*, *Oxyethira houailou* sp. n., *Oxyethira insularis*, *Oxyethira oropedion*, *Oxyethira melasma*, *Oxyethira digitata* sp. n., *Acritoptila disjuncta*, *Acritoptila crinita*, *Acritoptila ouenghica* Wells, 1995, *Caledonotrichia illiesi* Sykora, 1967, *Caledonotrichia minuta* Wells, Johanson & Mary-Sasal, 2013, *Caledonotrichia ouinnica* Wells, Johanson & Mary-Sasal, 2013, *Caledonotrichia nyurga* Oláh & Johanson, 2010, *Paroxyethira atypica* Wells & Johanson, 2012 and *Paroxyethira dzumac* Wells & Johanson, 2012) **80** *Oxyethira dorsennus* sp. n. (collected together with the hydroptilid species *Hydroptila losida* and *Paroxyethira dumagnes* Kelley, 1984).

**Figures 81–85.**
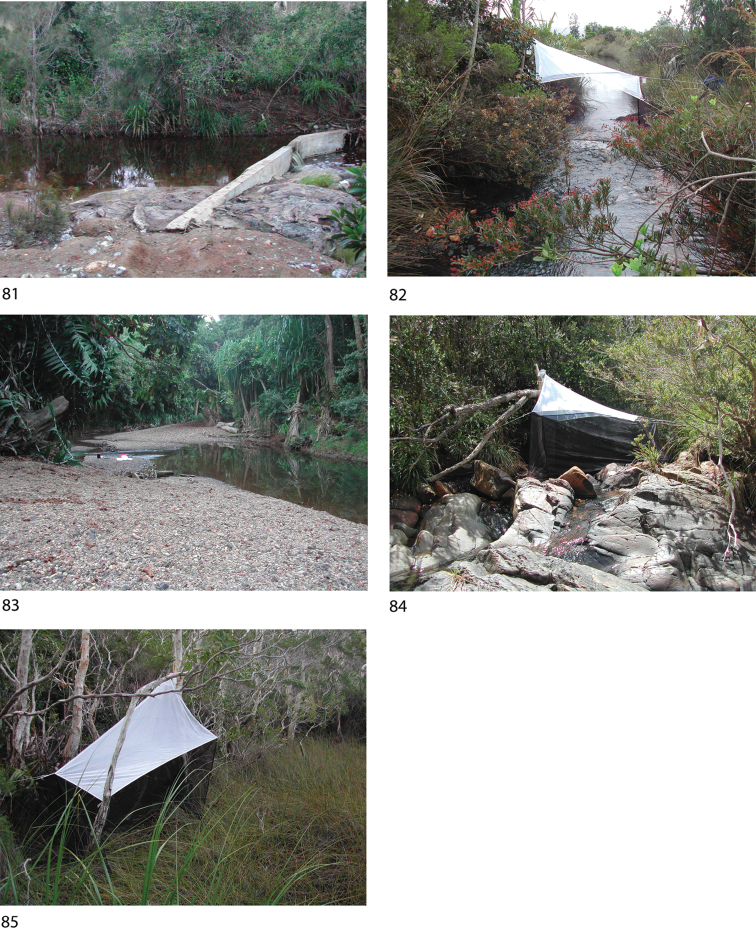
Type localities of *Oxyethira* species. **81** *Oxyethira rougensis* sp. n. (collected together with the hydroptilid species *Oxyethira oropedion*, *Hydroptila losida*, *Hellyethira malleoforma*, *Acritoptila crinita*, *Acritoptila macrospina* Wells & Johanson, 2014 and *Paroxyethira opposita* Wells & Johanson, 2012) **82** *Oxyethira enigmatica* sp. n. (collected together with the hydroptilid species *Oxyethira perignonica* sp. n., *Oxyethira melasma* and *Acritoptila disjuncta*) **83** *Oxyethira nehoue* sp. n. (collected together with the hydroptilid species *Oxyethira oropedion*, *Oxyethira incana*, *Hydroptila losida*, *Hellyethira malleoforma* and *Acritoptila disjuncta*) **84** *Oxyethira digitata* sp. n. (collected together with the hydroptilid species *Oxyethira incurvata* sp. n., *Oxyethira indorsennus*, *Oxyethira scutica*, *Hydroptila losida*, *Caledonotrichia illiesi* and *Caledonotrichia minuta*) **85** *Oxyethira macropennis* sp. n. (no other Hydroptilidae species collected at this site).

**Figures 86–93.**
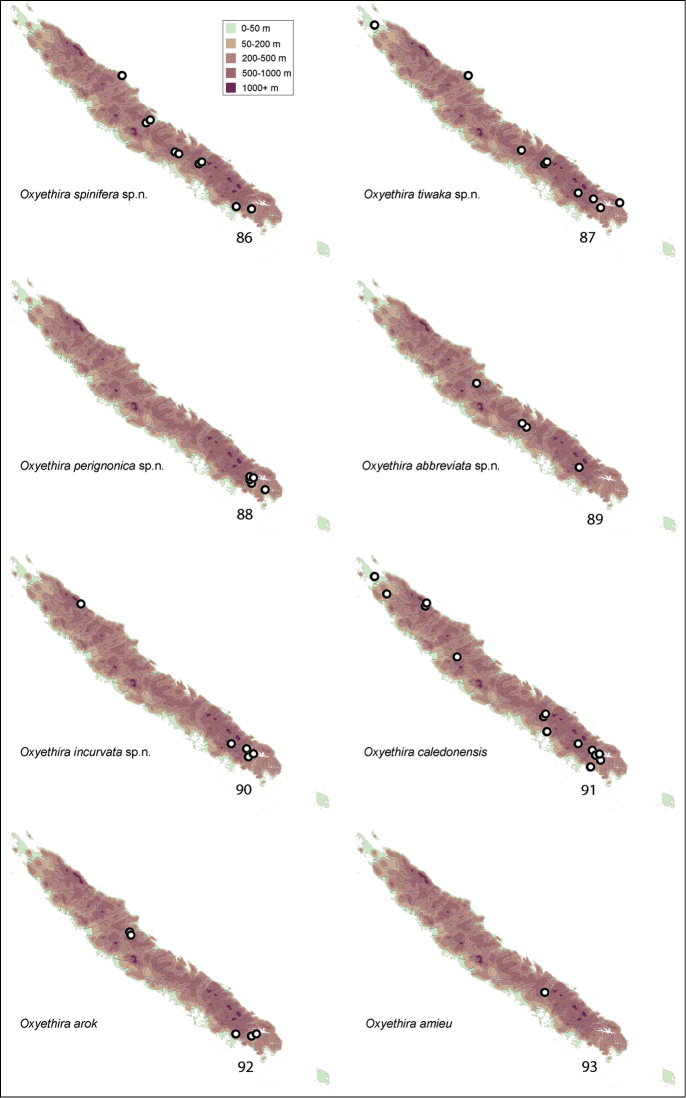
Maps of New Caledonia, with collecting sites plotted for Hydroptilidae species. **86** *Oxyethira spinifera* sp. n. **87** *Oxyethira tiwaka* sp. n. **88** *Oxyethira perignonica* sp. n. **89** *Oxyethira abbreviata* sp. n. **90** *Oxyethira incurvata* sp. n. **91** *Oxyethira caledonensis* **92** *Oxyethira arok* **93** *Oxyethira amieu* sp. n.

**Figures 94–101.**
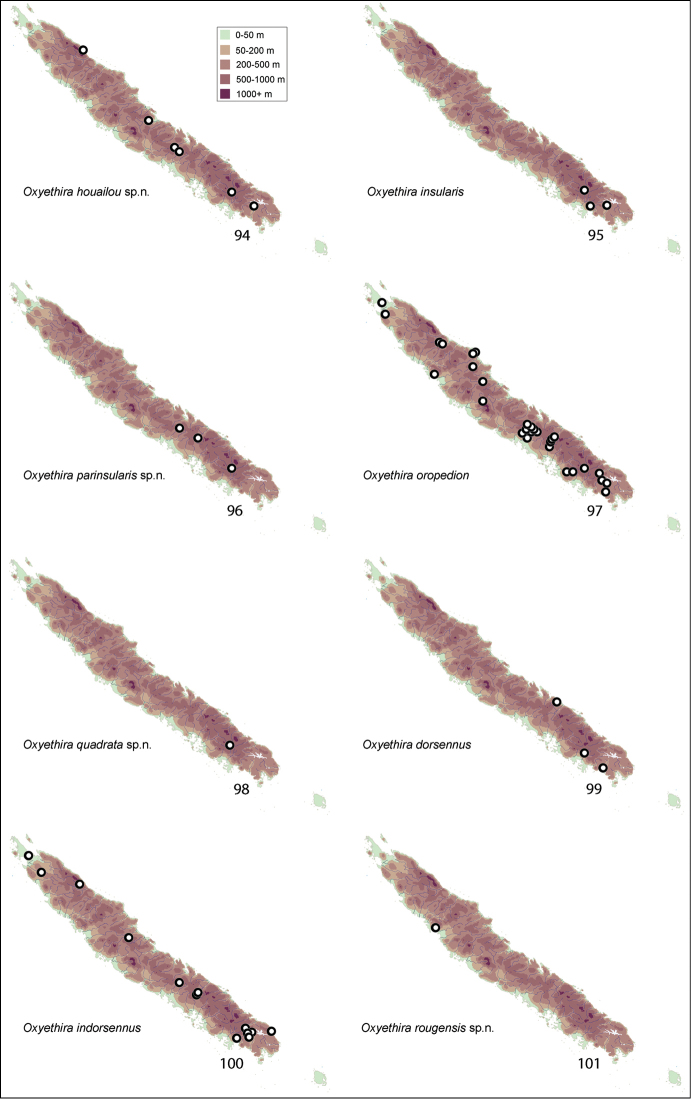
Maps of New Caledonia, with collecting sites plotted for Hydroptilidae species. **94** *Oxyethira houailou* sp. n. **95** *Oxyethira insularis* **96** *Oxyethira parinsularis* sp. n. **97** *Oxyethira oropedion* **98** *Oxyethira quadrata* sp. n. **99** *Oxyethira dorsennus* **100** *Oxyethira indorsennus* **101** *Oxyethira rougensis* sp. n.

**Figures 102–109.**
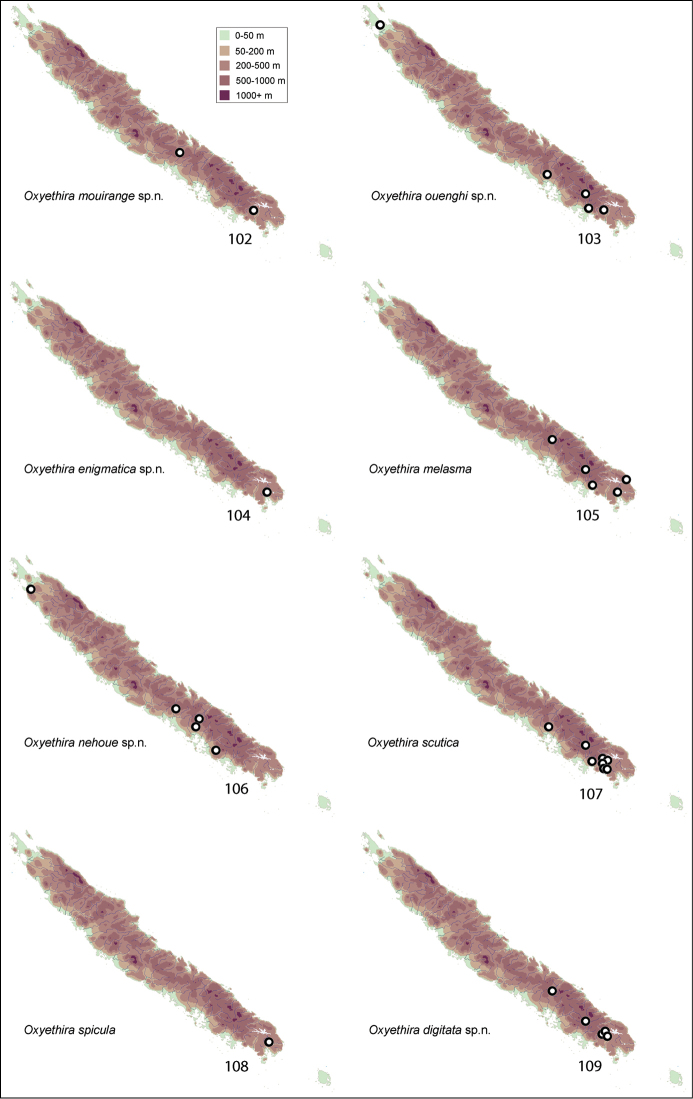
Maps of New Caledonia, with collecting sites plotted for Hydroptilidae species. **102** *Oxyethira mouirange* sp. n. **103** *Oxyethira ouenghi* sp. n. **104** *Oxyethira enigmatica* sp. n. **105** *Oxyethira melasma* **106** *Oxyethira nehoue* sp. n. **107** *Oxyethira scutica* **108** *Oxyethira spicula* sp. **109** *Oxyethira digitata* sp. n.

#### Subgenus *Dampfitrichia* Ulmer

Erected at genus level by Mosely (1937: p.169), and synonymised with *Oxyethira* by Ross (1944), *Dampfitrichia* was accorded subgenus status by Kelley (1984) in *Oxyethira* and diagnosed as “… characterised by a fusion of veins R4 and R5 in the forewing and the subdistal sclerotised bridge between the subgenital processes”; Kelley noted that the phallic apparatus usually lacks a titillator.

##### Oxyethira (Dampfitrichia) incana

Taxon classificationAnimaliaTrichopteraHydroptilidae

Ulmer

Figs 70 , 71 , 110

Oxyethira incana Ulmer, 1906: 102 (see Morse 2015 for full synonymy)

###### Diagnosis.

Males of this species are distinguished from others in the New Caledonian fauna by abdominal segment VIII with disto-lateral angles spiny, and venter produced and rounded distally not excised apico-ventrally, forming a shield over other genital structures which are strongly fused; and by phallic apparatus curiously stout and medially curved, lacking a titillator. Female terminalia longer and terminally more slender than those of *caledoniensis* group species, but not as slender as in members of subgenus *Pacificotrichia*, with sternite X bearing a jet black quadrate plate.

Antennae: males 25–28 flagellomeres, flagellomeres about twice as long as wide; female 20–21 flagellomeres, flagellomeres subquadrate in profile. Anterior wing length: males 1.8–2.2 mm (n=10); females 1.8–2.4 mm (n=10). Spurs 0,3,4. Abdominal sternite VII without median spine.

###### Material examined.

Numerous males, females, Province Nord, Amoa River, 23 m, loc 20, 12 km W Poindimié, 22°58.092'S, 165°11.804'E, light trap, 26.xi.2001, leg. K.A. Johanson, T. Pape & B. Viklund (NHRS, ANIC); 2 males, 2 females, Province Nord, 50 m upstream bridge on Hienghène–Tnèdo road, 3.9 km S summit of Mt Tnèda, 2.2 km E Tnèdo, 20°43.085'S, 164°49.928'E, 29 m, 7.xii.2003, light trap, loc#071, leg. K.A. Johanson (NHRS); numerous males, females, Province Nord, 1 m upstream road, below waterfall on Hienghène–Tnèdo road, 2.2 km SSW summit of Mt Unpac, 4.9 km ESE Tnèdo, 20.73879°S, 164.85508°E, 7.xii.2003, light trap, loc#072, leg. K.A. Johanson (NHRM); 3 males, 6 females, Province Nord, stream in Creek de Bambou, 5 m N road RT7 Ouégoa–Koumac, 20°27.863'S, 164°19.784'E, 58 m, 19.xii.2003, Malaise trap, loc#087, leg. K.A. Johanson (NHRS); numerous males, females, Province Nord, Bouérabate Stream, S Mont Ninndo, along road Barabache–Boulagoma, 20°17.409'S, 164°11.242'E, 60 m, 19.xii.2003–7.i.2004, Malaise trap, loc#089, leg. K.A. Johanson; 2 males, 4 females, Province Nord, Rivière Néhoué, camp Amenage de Néhoué, 20°25.037'S, 164°13.222'E, 12 m, 19.xii.2003, light trap, loc#090, leg. K.A. Johanson (NHRS); numerous males, females, Province Nord, Héémwâ Pwei River, 50 m upstream bridge on Touho–Hienghene road, 1.0 km N Paola, 20.76512°S, 165.10979°E, 22.xii.2003, light trap, loc#095, leg. K.A. Johanson (NHRM); 3 male, 6 females, Province Nord, Ponandou Tiôgé River at Kögi, 3.9 km SSW Touho, 20°49.043'S, 165°13.551'E, 25 m, 26.xii.2003, light trap, loc#100, leg. K.A. Johanson (NHRS).

###### Remarks.

**Figures 110–113.**
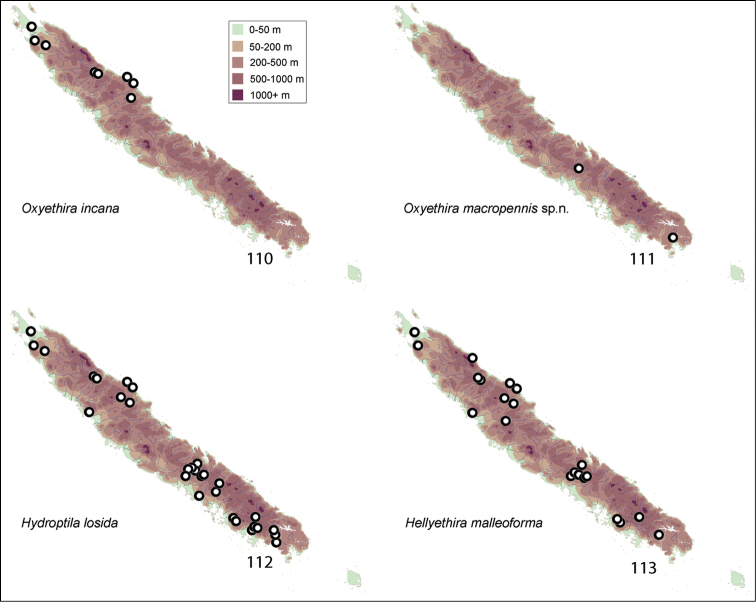
Maps of New Caledonia, with collecting sites plotted for Hydroptilidae species. **110** *Oxyethira incana* **111** *Oxyethira macropennis* sp. n. **112** *Hydroptila losida* sp. n. **113** *Hellyethira malleoforma*.

In New Caledonia this species was taken only in the northern province (Fig. 110); elsewhere it is widespread from Java, through South-East Asia to New Guinea and northern Australia.

#### *Oxyethira* species unplaced to subgenus.

One highly aberrant species, *Oxyethira macropennis* sp. n., is here unplaced to subgenus. Males share the diagnostic features of species of *Oxyethira*, including wing shape and venation, but have unusual male genital features, possibly aligning the species more with species of *Paroxyethira*. For the present we assign it to *Oxyethira*, albeit tentatively.

##### Oxyethira macropennis sp. n.

Taxon classificationAnimaliaTrichopteraHydroptilidae

http://zoobank.org/3FA585C6-F464-4B22-B39E-C3B1172F5A0C

Figs 72–74 , 85 , 111

###### Diagnosis.

Immediately recognised by the remarkable form of the phallic apparatus, which has a contorted twist towards the stout seta-bearing apex, and the brush of shorter stout setae medially on abdominal sternite IX. By these features it is distinguished clearly from all other New Caledonian species.

###### Description.

Male antennae with 22–24 flagellomeres; flagellomeres urn-shaped, few *sensilla placodea* subapically, dense *sensilla auricillica*; anterior wing length 1.7–2.1 mm (n=5); tibial spurs 0,3,4; abdominal sternite VII without medial spur.

Male, genitalia (Figs 72–74). Abdominal segment VIII quadrate in profile; abdominal segment IX subrectangular, ventrally bearing a brush of stout setae medially, and on each side a cluster of shorter setae subapically, mid dorsally short, apical margin shallowly excavated; gonopods may be represented by the sclerotised apico-lateral lobes on abdominal segment IX; phallic apparatus strongly twisted in distal half, beyond a short lateral process that may represent titillator, subapically irregular in shape, bearing a stout seta.

###### Remarks.

*Oxyethira macropennis* is quite unlike all other New Caledonian species, however it conforms with the diagnostic features of members of the *Oxyethira* and thus is assigned to this genus, albeit somewhat tentatively.

###### Material examined.

**Holotype.** Male (on slide), New Caledonia, Province Sud, south of Plaine des Lacs, 4.0 km N Prony, 22°16.906'S, 166°49.402'E, 9–22.xi.2003, Malaise trap, loc#017, leg. K.A. Johanson (MNHP).

**Paratypes.** 6 males (2 on slides), data as for holotype (NMHR); 1 male, Province Sud, Sarraméa, 220 m, forest stream, loc 10, 21°37.883'S 165°51.958'E, Malaise trap, 18–21.xi.2001, leg. K.A. Johanson, T. Pape & B. Viklund (NHRS).

###### Etymology.

Name descriptive of the relatively large phallic apparatus.

###### Remarks.

*Oxyethira macropennis* was taken only at two well-separated sites in the south of the island. A photograph of the type locality with the trap is rendered in Fig. 85.

### New records of other genera

#### Hydroptila losida

Taxon classificationAnimaliaTrichopteraHydroptilidae

Mosely

Fig. 112

Hydroptila losida Mosely, 1953: 505; Wells 1978 [1979]: 757, figs 35–38; Wells 1995: 231.

##### Diagnosis.

The only *Hydroptila* species among New Caledonian micro-caddisflies, *Hydroptila losida* is recognised by the absence of ocelli on the dorsal head, and tibial spur count of 0,2,4; and in the male by gonopods well developed, in ventral view elongate divergent rods, each with a pair of dark spurs distally, in lateral view, club-shaped; and phallic apparatus slender, distally comprising a slender, tapered spine adpressed to the section containing the ejaculatory duct, or in some specimens these two parts are separated and divergent; and in the female by the triangular shape of sternite VIII with the two small triangular sclerotised areas laterally at about half length of the sternite.

##### Material examined.

Numerous males, females, Province Nord, Amoa River, 23 m, loc 20, 12 km W Poindimié, 22°58.092'S, 165°11.804'E, light trap, 26.xi.2001, leg. K.A. Johanson, T. Pape & B. Viklund(NHRS); 6 females, Province Sud, Col d’Amieu, 323 m, small stony river, loc 24, 21°34.844'S, 165°49.677'E, Malaise trap, 30.xi–5.xii.2001, leg. K.A. Johanson, T. Pape & B. Viklund(NHRS); numerous males, females, Province Nord, 2.8 km ENE Bopope, Rivière Oua Mendiou, 100 m S RPN2 Koné–Poindimié, 20°54.455'S, 165°06.300'E, 78 m, 14.i.2003, light trap, loc#119, leg. K.A. Johanson (NHRS); numerous males, females, Province Sud, Dumbea river, Branche sud, 22°08.344'S, 166°30.147'E, 42 m, 03.xi.2003, light trap, loc#006, leg. K.A. Johanson (NHRS); 3 males, Province Sud, side stream to Rivière Blanche, 10.75 km SW Pont Pérignon, 22°10.073'S, 166°39.903'E, 180 m, 6–16.xi.2003, Malaise trap, loc#012, leg. K.A. Johanson (NHRS); numerous males, females, Province Sud, Monts Kwa Ne Mwa, on road between Noumea and Yaté, 2.0 km E Pic Mouirange, 22°12.356'S, 166°40.798'E, 220 m, 7–16.xi.2003, Malaise trap, loc#014, leg. K.A. Johanson (NHRS); 4 males, 12 females, Province Sud, Sarraméa, 220 m, forest stream, loc 10, 21°37.883'S 165°51.958'E, Malaise trap, 18–21.xi.2001, Leg. K.A. Johanson, T. Pape & B. Viklund; 1 male, 6 females, Province Sud, Sarraméa, 2907 m, stony forest stream, loc 13 21°37.097'S 165°49.351'E, Malaise trap, 18–21.xi.2001, Leg. K.A. Johanson, T. Pape & B. Viklund (RHNS); 1 male, 2 females, Province Sud, Mt Dzumac, source stream of Ouinne River, near crosspoint to mountain track, 22°02.439'S, 166°28.646'E, 805 m, 18.xi–4.xii.2003, Malaise trap, loc#029, leg. K.A. Johanson (NHRS); numerous males, females, Province Sud, Tamoa River, 700m S road RT1 between Noumea and La Foa, 22°04.518'S, 166°16.592'E, 19.xi.2003, light trap, loc#033, leg. K.A. Johanson (NHRS); numerous males, females, Province Sud, Hwa Hace Mtn, Hwa Motu River, at Pont Wamuttu, 1.0 km E Nassirah, about 200 m upstream bridge, 21°48.094'S, 166°04.298'E, 137 m, 20.xi–12.xii.2003, Malaise trap, loc#034, leg. K.A. Johanson (NHRS); 2 males, 8 females, New Caledonia, Province Sud, stream at Refuge de Farino, 4.0 km W Grand Couli village, 21°38.934'S, 165°46.845'E, 260 m, 25.xi.2003, light trap, loc#044, leg. K.A. Johanson (NHRS); 30 males, Province Sud, St. Vincent, Bongou Stream, at bridge on road to Tribu de Bangou, 700 m N RT1 Noumea–Tontoutu road, 22°03.477'S, 166°15.718'E, 26.xi.2003, light trap, loc#050, leg. K.A. Johanson (NHRS); numerous males, females, Province Sud, Couvelée River at Haute Couvelée, 2.8 km SV summit of Mt Piditéré, 3.5 km NNE Dumbéa, 22°07.405'S, 166°28.023'E, 27 m, 28.xi.2003, light trap, loc#052, leg. K.A. Johanson (NHRS); 3 females, Province Sud, W slope Mt Ningua, Kwé Néco, Stream, at Camp Jacob, 3.7 km WNW summit of Mt Ningua, on Boulouparis–Thio Road, about 50 m upstream road, 21°43.613'S, 166°06.567'E, 150 m, 29.xi–12.xii.2003, Malaise trap, loc#054, leg. K.A. Johanson (NHRS); 4 males, 12 females, Province Sud, lower part of Dumbea River, 1.0 km SSW bridge over Dumbea River at Dumbea, 22°09.750'S, 166° 26.700'E, 0.5 m, 30.xi.2003, light trap, loc#058, leg. K.A. Johanson (NHRS); 1 male, 6 female, Province Sud, lower part Rivière des Pirogues, 800 m WNW summit of Mont Imbaah, 4.7 km E Lucky Creek in Plum, 22°18.559'S, 166°41.227'E, 1.3 m, 01.xii.2003, light trap, loc#059, leg. K.A. Johanson (NHRS); 6 males, 4 females, Province Nord, 50 m upstream bridge on Hienghène–Tnèdo road, 3.9 km S summit of Mt Tnèda, 2.2 km E Tnèdo, 20°43.085'S, 164°49.928'E, 29 m, 7.xii.2003, light trap, loc#071, leg. K.A. Johanson (NHRS); numerous males, females, Province Nord, 1 m upstream road, below waterfall on Hienghène–Tnèdo road, 2.2 km SSW summit of Mt Unpac, 4.9 km ESE Tnèdo, 0.73879°S, 164.85508°E, 7.xii.2003, light trap, loc#072, leg. K.A. Johanson (NHRS); 1 male, 3 females, Province Nord, stream in Creek de Bambou, 5 m N road RT7 Ouégoa–Koumac, 20°27.863'S, 164°19.784'E, 58 m, 19.xii.2003, Malaise trap, loc#087, leg. K.A. Johanson (NHRS); 3 males, Province Nord, Bouérabate Stream, S Mont Ninndo, along road Barabache–Boulagoma, 20°17.409'S, 164°11.242'E, 60 m, 19.xii.2003–7.i.2004, Malaise trap, loc#089, leg. K.A. Johanson (NHRS); numerous males, females, Province Nord, Rivière Néhoué, camp Amenage de Néhoué, 20°25.037'S, 164° 13.222'E, 12 m, 19.xii.2003, light trap, loc#090, leg. K.A. Johanson (NHRS); numerous males, females, Province Nord, Rivière Néhoué, camp Amenage de Néhoué, 20°25.015'S, 164°13.245'E, 12 m, 19.xii.2003, light trap, loc#091, leg. K.A. Johanson (NHRS); numerous males, females, Province Nord, Héémwâ Pwei River, 50 m upstream bridge on Touho–Hienghene road, 1.0 km N Paola, 20.76512°S, 165.10979°E, 22.xii.2003, light trap, loc#095, leg. K.A. Johanson (NHRS); numerous males, females Province Nord, Ponandou Tiôgé River at Kögi, 3.9 km SSW Touho, 20°49.043'S, 165°13.551'E, 25 m, 26.xii.2003, light trap, loc#100, leg. K.A. Johanson (NHRS); males, females, Province Nord, Plaine des Gaïacs, Rivière Rouge, 14.2 km NW summit of Mt Rouge, 50 m upstream road RT1 Noumea–Koné, 21°31.573'S, 164°46.690'E, 23 m, 2.i.2004, light trap, loc#104, leg. K.A. Johanson (NHRS); 1 male, Province Sud, Creek Froid, 10 m upstream bridge on La Foa–Koindé road, 200 m W crossroad to Ouipouin, 21°38.581'S, 165°56.672'E, 180 m, 4.i.2004, light trap, loc#105, leg. K.A. Johanson (NHRS); 3 males, Province Sud, Fö Néchédeva stream, 2 m upstream bridge on La Foa–Koindé road, 21°38.812'S, 165°56.076'E, 124 m, 4.i.2004, light trap, loc#106, leg. K.A. Johanson (NHRS); numerous males, females, Province Nord, Etnbl. thermal de la Crouen, along Riv. la Crouen, 30 m upstream road RM3, 21°32.105'S, 165°53.319'E, 15 m, 5.i.2004, Malaise trap, loc#110, leg. K.A. Johanson (NHRS); 1 male, 5 females, Province Sud, Xwê Dachava Stream, Rembai Mtn, 21°34.854'S, 165°49.478'E, 317 m, 5–12.i.2004, Malaise trap, loc#108, leg. K.A. Johanson (NHRS); 11 males, Province Sud, Col d’Amieu, Xwé Ko River, on road to St. Forestière, 21°35.612'S, 165° 48.241'E, 368 m, 8.i.2004, light trap, loc#114, leg. K.A. Johanson (NHRS); numerous males, females, Province Sud, Sarraméa, Xwê Wya River, 21°38.318'S, 165°51.582'E, 127 m, 17–18.i.2004, light trap, loc#121, leg. K.A. Johanson (NHRS); 2 males, numerous females, Province Sud, artificial lake 2.6 km S summit of Mt Mè Tu Novia, about 400 m N Pocquereux River, 7.4 km E La Foa, 21°43.859'S, 165°54.034'E, 28 m, 19.i.2004, light trap, loc#123, leg. K.A. Johanson (NHRS).

##### Remarks.

In New Caledonia *Hydroptila losida* is abundant and widespread across the island (Fig. 112). A similar wide distribution is true of this species in eastern Australia where it is common throughout the wetter coastal regions, including the south-west and Tasmania, but not the far north of the continent.

#### Hellyethira malleoforma

Taxon classificationAnimaliaTrichopteraHydroptilidae

Wells

Fig. 113

Hellyethira malleoforma Wells, 1979: figs 41–45; Wells 1995: 232.

##### Diagnosis.

Males of this species are distinguished by their complex asymmetrical genital structures, including multilobed gonopods, and females by the sclerotised annulus formed by abdominal segment VIII (see Wells 1979).

##### Material examined.

2 males, females, Province Sud, Sarraméa, 220 m, forest stream, loc 10, 21°37.883'S 165°51.958'E, Malaise trap, 18–21.xi.2001, leg. K.A. Johanson, T. Pape & B. Viklund(NHRS); 1 male, 4 females, Province Sud, Sarraméa, 2907 m, stony forest stream, loc 13, 21°37.097'S 165°49.351'E, Malaise trap, 18–21.xi.2001, leg. K.A. Johanson, T. Pape & B. Viklund(NHRS); numerous males, females, Province Nord, Amoa River, 23 m, loc 20, 12 km W Poindimié, 22°58.092'S, 165°11.804'E, light trap, 26.xi.2001, leg. K.A. Johanson, T. Pape & B. Viklund(NHRS); 1 male, 4 females, Province Sud, Monts Kwa Ne Mwa, on road between Noumea and Yaté, 2.0 km E Pic Mouirange, 22°12.356'S, 166°40.798'E, 220 m, 7–16.xi.2003, Malaise trap, loc#014, leg. K.A. Johanson (NHRS); 2 males, 17 females, Province Sud, Mt Dzumac, source stream of Ouinne River, near crosspoint to mountain track, 22°02.439'S, 166°28.646'E, 805 m, 18.xi–4.xii.2003, Malaise trap, loc#029, leg. K.A. Johanson (NHRS); males, females, Province Sud, Tamoa River, 700m S road RT1 between Noumea and La Foa, 22°04.518'S, 166°16.592'E, 19.xi.2003, light trap, loc#033, leg. K.A. Johanson (NHRS); males, females, Province Sud, stream at Refuge de Farino, 4.0 km W Grand Couli village, 21°38.934'S, 165°46.845'E, 260 m, 25.xi.2003, light trap, loc#044, leg. K.A. Johanson (NHRS); 4 males, 2 females, St. Vincent, Bongou Stream, at bridge on road to Tribu de Bangou, 700 m N RT1 Noumea–Tontoutu road, 22°03.477'S, 166°15.718'E, 26.xi.2003, light trap, loc#050, leg. K.A. Johanson (NHRS); numerous males, females, Province Nord, 50 m upstream bridge on Hienghène–Tnèdo road, 3.9 km S summit of Mt Tnèda, 2.2 km E Tnèdo, 20°43.085'S, 164°49.928'E, 29 m, 7.xii.2003, light trap, loc#071, leg. K.A. Johanson (NHRS); males, females, Province Nord, 1 m upstream road, below waterfall on Hienghène–Tnèdo road, 2.2 km SSW summit of Mt Unpac,4.9 km ESE Tnèdo, 20.73879°S, 164.85508°E, 7.xii.2003, light trap, loc#072, leg. K.A. Johanson (NHRS); males, females, Province Nord, Wan Pwé on stream, draining NNE side of Mt Panié, 3.9 km NW Cascade de Tao, 20°31.820'S, 164°47.016'E, 18.xii.2003, light trap, loc#085, leg. K.A. Johanson (NHRS); numerous males, females, Province Nord, Bouérabate Stream, S Mont Ninndo, along road Barabache–Boulagoma, 20°17.409'S, 164°11.242'E, 60 m, 19.xii.2003–7.i.2004, Malaise trap, loc#089, leg. K.A. Johanson (RHNS); males, females, Province Nord, Rivière Néhoué, camp Amenage de Néhoué, 20°25.037'S, 164° 13.222'E, 12 m, 19.xii.2003, light trap, loc#090, leg. K.A. Johanson (NHRS); males, females, Province Nord, Rivière Néhoué, camp Amenage de Néhoué, 20°25.015'S, 164° 13.245'E, 12 m, 19.xii.2003, light trap, loc#091, leg. K.A. Johanson (NHRS); male, females, Province Nord, Héémwâ Pwei River, 50 m upstream bridge on Touho–Hienghene road, 1.0 km N Paola, 20.76512°S, 165.10979°E, 22.xii.2003, light trap, loc#095, leg. K.A. Johanson (NHRS); numerous males, females, Province Nord, Ponandou Tiôgé River at Kögi, 3.9 km SSW Touho, 20°49.043'S, 165°13.551'E, 25 m, 26.xii.2003, light trap, loc#100, leg. K.A. Johanson (NHRS); males, females, Province Nord, Plaine des Gaïacs, Rivière Rouge, 14.2 km NW summit of Mt Rouge, 50 m upstream road RT1 Noumea–Koné, 21°31.573'S, 164°46.690'E, 23 m, 2.i.2004, light trap, loc#104, leg. K.A. Johanson (NHRS); 1 male, KAJ sp. F, Province Sud, Creek Froid, 10 m upstream bridge on La Foa–Koindé road, 200 m W crossroad to Ouipouin, 21°38.581'S, 165°56.672'E, 180 m, 4.i.2004, light trap, loc#105, leg. K.A. Johanson (NHRS); 1 male KAJ sp. F, Province Sud, Fö Néchédeva stream, 2 m upstream bridge on La Foa–Koindé road, 21°38.812'S, 165°56.076'E, 124 m, 4.i.2004, light trap, loc#106, leg. K.A. Johanson (NHRS); numerous males, females, Province Nord, Etnbl. thermal de la Crouen, along Riv. la Crouen, 30 m upstream road RM3, 21°32.105'S, 165°53.319'E, 15 m, 5.i.2004, Malaise trap, loc#110, leg. K.A. Johanson 1 male KAJ “F”, Province Nord, Forêt Plate, Ouendé River, at 2.5 km WNW summit of Katépouenda, 23.3 km E Pouembout, 21°07.474'S, 165°06.781'E, 470 m, 8–15.i.2004, Malaise trap, loc#113, leg. K.A. Johanson (NHRS); numerous males, females, Province Nord, 2.8 km ENE Bopope, Rivière Oua Mendiou, 100 m S RPN2 Koné–Poindimié, 20°54.455'S, 165°06.300'E, 78 m, 14.i.2003, light trap, loc#119, leg. K.A. Johanson (NHRS).

##### Remarks.

*Hellyethira malleoforma* is the only representative in New Caledonia of this diverse Australian genus that also occurs more broadly but less commonly in SE Asia and New Guinea. This species is widespread and often abundant in New Caledonia (Fig. 113). It was described from south-eastern Australia where it is one of the most common species in lower altitude streams.

### Key to males of New Caledonian species of *Oxyethira*

**Table:** 

1	Tibial spur formula 0,2,4	**2**
–	Tibial spur formula 0,3,4	**6**
2(1)	Phallic apparatus tipped by fine whip-like flagellum (Fig. 61)	***Oxyethira scutica***
–	Phallic apparatus with strap-like distal process (Figs 7, 53), or without process (Figs 40, 57)	**3**
3(2)	Phallic apparatus with strap-like apical or subapical process (Figs 8, 53)	**4**
–	Phallic apparatus without apical process (Figs 40, 57)	**5**
4(3)	Phallic apparatus with elongate strap-like apical process (Fig. 53); abdominal segment IX in ventral view distally bilobed (Fig. 53)	***Oxyethira enigmatica***
–	Phallic apparatus with short strap-like subapical process (Fig. 8); abdominal segment IX in ventral view subquadrate (Fig. 7)	***Oxyethira perignonica***
5(3)	Gonopods fused at base, separate distally, apices truncate in ventral view (Fig. 40); ventral process sharply triangular in lateral view (Fig. 39)	***Oxyethira dorsennus***
–	Gonopods fused throughout, narrowly truncate apically, a pair of small setose lobes at mid length (Fig. 55); ventral process broadly rounded to truncate in lateral view (Fig. 56)	***Oxyethira melasma***
6(1)	Phallic apparatus twisted and irregular in shape distally, bearing a single stout seta (Fig. 74)	***Oxyethira macropennis***
–	Phallic apparatus without seta, with strap-like flange or process (e.g. Figs 11, 30), or simple without apical or subapical processes (e.g. Figs 57, 71)	**7**
7(6)	Gonopods inserted midway or proximally on venter of abdominal segment IX, in ventral view in form of Y-shaped structure or pair of widely separated ‘horns’ (Figs 28, 32), in lateral view in form of curved spines (Fig. 33)	**8**
–	Gonopods situated distally on venter of abdominal segment IX (e.g. Figs 10, 25, 70) or completely reduced (e.g. Figs 1, 64)	**9**
8(7)	Gonopods in ventral view distinctly Y-shaped (Fig. 28)	***Oxyethira insularis***
–	Gonopods in ventral view in form of pair of widely separated spines joined basally by short sclerotised strip (Fig. 32)	***Oxyethira parinsularis***
9(7)	Abdominal segment VIII extended disto-laterally as pair of sclerotised spines (Fig. 70)	***Oxyethira incana***
–	Abdominal segment VIII without apico-lateral sclerotised spines (Figs 1, 4, 10)	**10**
10(9)	Ventral processes in form of pair of laterally situated rods or spines (Figs 1, 4, 10)	**11**
–	Ventral processes fused, in ventral view forming median plate (Figs 36, 38, 40, 44)	**18**
11(10)	Abdominal segment IX in ventral view subquadrate (Figs 2, 4, 10)	**12**
–	Abdominal segment IX in ventral view rounded, conical or triangular proximally (Figs 13, 19, 23)	**14**
12(11)	Gonopods forming sclerotised cones at apico-lateral angles of abdominal segment IX (Fig. 10)	***Oxyethira abbreviata***
–	Gonopods reduced completely or in form of short blunt tabs, widely separated on apical margin of abdominal segment IX (Figs 1, 4)	**13**
13(12)	Gonopods reduced completely (Fig. 1); ventral processes acute apically (Figs 1, 2)	***Oxyethira spinifera***
–	Gonopods in ventral view in form of blunt sclerotised tabs scarcely longer than wide, well separated on apical margin of abdominal segment IX (Fig. 4); ventral processes not as sharp as in *Oxyethira spinifera* (Figs 4, 6)	***Oxyethira tiwaka***
14(10)	Gonopods apparently reduced completely or possibly present as marginal sclerotisations on distal margin of abdominal segment IX (Figs 19, 20, 23)	**15**
–	Gonopods recognisable as sclerotised prominences or processes on distal margin of abdominal segment IX (Figs 13, 16, 17, 25)	**16**
15(14)	Abdominal segment IX in ventral view tapered distally (Fig. 23); ventral processes sharply pointed in lateral view (Fig. 24); strap-like process subapical on phallic apparatus (Fig. 23)	***Oxyethira amieu***
–	Abdominal segment IX in ventral view parallel-sided in distal half (Figs 19, 20); ventral processes bluntly rounded apically in lateral view (Fig. 21); strap-like process subapical on phallic apparatus (Figs 19, 22)	***Oxyethira arok***
16.(14)	Gonopods in form of short domes situated slightly laterally on distal margin of abdominal segment IX (Figs 16, 17)	***Oxyethira caledoniensis***
–	Gonopods in ventral view broad, stoutly quadrate and separated by narrow v-shaped cleft (Fig. 25) or slender, laterally situated and curving mesially (Fig. 13)	**17**
17(16)	Gonopods in ventral view stoutly quadrate (Fig. 25)	***Oxyethira houailu***
–	Gonopods in ventral view in form of laterally situated finger-like mesally curved processes (Fig. 13)	***Oxyethira incurvata***
18(10)	Phallic apparatus dilated distally, with a sharp, sclerotised straight apical spine (Fig. 64); abdominal segment VIII completely obscuring gonopods and other genital processes (Fig. 64)	***Oxyethira spicula***
–	Phallic apparatus not as above; abdominal segment VIII not completely obscuring gonopods and other genital processes (e.g. Figs 38, 44, 68)	**19**
19(18)	Gonopods completely fused, in ventral view in form of triangular plate; ventral process in lateral view a stoutly sclerotised arch (Fig. 58)	***Oxyethira nehoue***
–	Gonopods either not fused or only fused basally (e.g. Figs 36, 38, 44, 51)	**20**
20(19)	Gonopods in ventral view branched, digitiform (Fig. 68)	***Oxyethira digitata***
–	Gonopods unbranched (e.g. Figs 36, 43 )	**21**
21(20)	Gonopods elongate triangular, acute apically (Figs 44, 47)	**22**
–	Gonopods in ventral view rounded apically (Figs 36, 38, 43, 44)	**23**
22(21)	Plate formed by fusion of subgenital processes subtriangular, slender in distal half, with paired short setae subapically (Fig. 44)	***Oxyethira ouenghica***
–	Plate formed by fusion of subgenital processes broadly triangular, rounded apically and without setae (Fig. 47)	***Oxyethira mouirange***
23(22)	Abdominal segment VIII, in ventral view, with medial cleft on distal margin narrow, deeper than wide (Fig. 44)	***Oxyethira rougensis***
–	Abdominal segment VIII, in ventral view, with wide U- or V-shaped excavation on distal margin, width greatly exceeding depth (Figs 36, 38, 43)	**24**
24(23)	Fused subgenital processes in ventral view in form of subquadrate plate (Fig. 38)	***Oxyethira quadrata***
–	Fused subgenital processes in ventral view tapered or rounded distally (Figs 36, 43)	**25**
25(24)	Gonopods in ventral view fused basally, widely separated distally pair of small membranous lobes in mid ventral position, each bearing a pale stout seta (Fig. 36)	***Oxyethira oropedion***
–	Gonopods in ventral view fused basally, free but closely abutting, with only sharp median cleft separation; without pair of median setal lobes (Fig. 43)	***Oxyethira indorsennus***

## Supplementary Material

XML Treatment for Oxyethira (Trichoglene) spinifera

XML Treatment for Oxyethira (Trichoglene) tiwaka

XML Treatment for Oxyethira (Trichoglene) perignonica

XML Treatment for Oxyethira (Trichoglene) abbreviata

XML Treatment for Oxyethira (Trichoglene) incurvata

XML Treatment for Oxyethira (Trichoglene) caledoniensis

XML Treatment for Oxyethira (Trichoglene) arok

XML Treatment for Oxyethira (Trichoglene) amieu

XML Treatment for Oxyethira (Trichoglene) houailou

XML Treatment for Oxyethira (Trichoglene) insularis

XML Treatment for Oxyethira (Trichoglene) parinsularis

XML Treatment for Oxyethira (Pacificotrichia) oropedion

XML Treatment for Oxyethira (Pacificotrichia) quadrata

XML Treatment for Oxyethira (Pacificotrichia) dorsennus

XML Treatment for Oxyethira (Pacificotrichia) indorsennus

XML Treatment for Oxyethira (Pacificotrichia) rougensis

XML Treatment for Oxyethira (Pacificotrichia) mouirange

XML Treatment for Oxyethira (Pacificotrichia) ouenghi

XML Treatment for Oxyethira (Pacificotrichia) enigmatica

XML Treatment for Oxyethira (Pacificotrichia) melasma

XML Treatment for Oxyethira (Pacificotrichia) nehoue

XML Treatment for Oxyethira (Pacificotrichia) scutica

XML Treatment for Oxyethira (Pacificotrichia) spicula

XML Treatment for Oxyethira (Pacificotrichia) digitata

XML Treatment for Oxyethira (Dampfitrichia) incana

XML Treatment for Oxyethira macropennis

XML Treatment for Hydroptila losida

XML Treatment for Hellyethira malleoforma
